# Supplementary material for: Plasmonic coffee-ring biosensing for AI-assisted point-of-care diagnostics
Source: Nat Commun. 2025 May 17;16:4597. doi: 10.1038/s41467-025-59868-y (PMC12085679; doi:10.1038/s41467-025-59868-y)
Supplement: Supplementary file 1 — Supplementary Information [file 41467_2025_59868_MOESM1_ESM.pdf]

Supplementary Information

Plasmonic Coffee-Ring Biosensing for AI-Assisted Point-of-Care Diagnostics

Kamyar Behrouzi[1,2,*], Zahra Khodabakhshi Fard[3], Chun-Ming Chen[1], Peisheng He[1,2], Megan Teng[1,2], Liwei Lin[1,2,*]

[1]: Department of Mechanical Engineering, University of California, Berkeley, CA, USA, 94720

[2]: Berkeley Sensor and Actuator Center (BSAC), Berkeley, CA, USA, 94720

[3]: Department of Applied Science and Technology, University of California, Berkeley, CA, USA, 94720

[*]: Corresponding Authors: kbehrouzi@berkeley.edu, lwlin@berkeley.edu

LOD Definitions

To assess and compare the sensitivity of biosensors, we applied suitable definitions for each case. For LFIA, we calculated $I = \left|\frac{I_T - I_B}{I_C - I_B}\right|$, where I is the gray index intensity; T, C, and B represent the test line, control line, and background, respectively. We then fitted Hill's model, $\frac{\theta_{Max} X^h}{K_d{}^h + X^h}$, to the I-versus-concentration data, where θ_{Max} is the saturated response at the maximum concentration; X is the concentration; h is the hill coefficient; and K_d is the concentration correspond to half-maximum binding signals [16]. The LOD was defined as the point where the fitted curve intersects with the constant intensity $I_{LOD} = I_{CS} + 3\sigma_{CS}$, where I_{LOD} is the LFIA response at LOD; I_{CS} is the response at the control sample; and σ_{CS} is the standard deviation of the control sample.

For the neural network-based detection (see **Figure 5e and i**), we employed two different definitions to characterize the biosensor LOD. The first method, called standard LOD, identifies the smallest concentration at which all predicted probabilities (e.g., for 10 instances in our case) of the input image remain above 0.5, as outlined in a prior research report [45]. This definition aligns with the limit of quantification (LOQ). We also introduced an alternative definition, called probabilistic LOD, based on the activation function in the final layer of the VGG-16 model (classification section). VGG-16 utilizes the sigmoid function, $P_S(x) = \frac{1}{1+e^{-x}}$, to calculate classification probabilities, so we applied the generalized sigmoid form (logistic regression), $P_{LR}(x) = \frac{1}{1+e^{-(b_0+b_1 x)}}$, and fitted this to the predicted probability data versus concentration. We defined the LOD as the intersection of the fitted curve with the constant probability line $P_{LOD} = 0.5 + 3\sigma_N$, where P_{LOD} is the estimated probability at LOD and σ_N is the maximum standard deviation at the negative zone. This definition captures the potential sensitivity of the biosensor, which is lower than the LOQ and similar to LFIA and other physics-based LOD definitions.

Numerical Modeling

Electromagnetic field distribution (**Figure 5c**) and optical absorption spectrum (**Figure 5d**) for specific and non-specific aggregated GNPs and GNShs have been modeled by the finite element method (FEM) using COMSOL Multiphysics software (V.6.1). To simplify the numerical model, periodic arrays of GNP or GNSh placed on top of the hydrophilic PTFE substrate have with an effective refractive index of 1.37 (considering the membrane porosity) have been used and the background refractive index is set to 1 as air (see **Figure S7**). The Floquet boundary condition was used to represent periodicity in solving the Maxwell's equation and perfectly matched layer (PML) on top and bottom boundaries were used (refer to **Figure S7**). The box height was larger than half of the maximum wavelength in each material and the mesh size was optimized for dielectric and plasmonic materials. The Lorentz-Drude model was chosen to represent plasmonic material (gold) [88]. To model specific and non-specific aggregates, we manipulated the periodicity of the unit cell in a way that the density of particles represents the specificity of the aggregates: tightly pack array for the non-specific form (large aggregates) and dispersed nanoparticles for the specific form (2D-like dispersed pattern) (see **Figure S7**). The optical absorption is normalized by the number of nanoparticles in the same 2D area.

Deep Neural Network Training

All machine learning algorithms were implemented using the TensorFlow library in Python. For CNN-based detection of positive versus negative samples, cropped grayscale images of the detection zone were used as inputs for training the network with positive and negative class labels (see **Figure 5, S8**). Experiments were carried out at varying concentrations for four different proteins, repeated multiple times, resulting in a dataset of approximately 100 unique images. This dataset was expanded using augmentation techniques such as rotation (see **Figure S26**) to create a sufficiently large amount of data for training the deep neural network. Even with the augmentation for a robust model, a dataset on the scale of thousands of images is typically required. To address this challenge, we employed transfer learning scheme by fine-tuning a pre-trained VGG-16 network, which was initially trained on the ImageNet dataset containing around 12 million images [76]. Both VGG-16 and ResNet-50 (different CNN architectures) have been used for similar classification tasks [89]. It is found that VGG-16 reaches lower loss values (binary cross-entropy) for higher accuracies at the same iteration number (see **Figure S15**, similar to the presented metrics in [90]). As such, the model was modified by adjusting the last layer from 1000 nodes to 256 and replacing the softmax function with a sigmoid function as our task was a binary classification. The model outputs a probability between 0 and 1 and values below 0.5 are considered as negative, while those above 0.5 are considered as positive.

For the C-GAN based segmentation of the specific zone, the Pix2Pix algorithm is utilized with grayscale detection zone images as inputs and manually extracted specific zones as the labels. The detailed structure of the network is depicted in **Figure S10 and 11**. Generative and discriminator networks are trained in tandem as shown in **Figure S12 and S13**, and the training is stopped when the generated specific zone and the ground truth are similar, since loss values are not a reliable source of action in training the generative networks [91]. All images are normalized prior to training and their sizes are 256 by 256 pixels.

The algorithm to quantify concentration value uses extracted lines to cross the plasmonic residual droplet coffee-ring around the center to increase the training data. The simple FC network is used to replace the non-linear relationship between the hidden information in each image and the sample concentration (the detailed network architecture in **Figure S14**). Since the crossline sizes are not the same for all samples, we fixed the size and attached constant 1 to the extended part, resampling white region (255 in grayscale, 1 in normalized scale) outside the coffee-ring region. The random sampling and flipping augmentation are crucial for getting reliable predictions from the network. The networks training process, test, and evaluation have been done on Google Colab. Information about loss variations with training epochs is provided in **Figure S15**. Additionally, we evaluated the performance of the model under various conditions, including lighting variations and different levels of imaging sensor noises (see **Figure S24-25**). These analyses revealed minimal variations to highlight the robustness of the model. It is noted that the tilt angle variations can be restored by scaling back the detection zone image into the circular shape (see **Figure S26**).

Table.S1: Comparing different biosensors for N-Protein of SARS-CoV-2 detection. We fixed the biomarker to make a fair comparison. This table includes some state-of-the art point-of-care (POC) biosensors, plus a few standard methods for comprehensive comparison. Note, Naked Eye means that the method does not require any special tool to determine the signal and the signal is visually clear, however post-processing can be applied to make the detection process easier.

Article/Company	Type	Instrument	Response Time	LOD
**Gupta et al.** [1]	Colorimetric (p-LFA)	Naked Eye	~20 min	76 ng/ml
**Gupta et al.** [1]	Fluorescent (p-LFA)	Fluorescent Reader	~20 min	212 pg/ml
**Gupta et al.** [1]	P-FLISA	Fluorescent Reader	~4 hr	102 pg/ml
**Cho et al.** [2]	Electrochemical	Impedance Analyzer (ToAD)	~1 hr	100 pg/ml
**Nicollete et al.** [3]	Enhanced LFIA	Imagin System (HiLab)	~15 min	2 ng/ml
**Shen et al.** [4]	Conventional LFIA	Naked Eye	~13 min	25 ng/ml
**Quanterix**	SiMoA	SiMoA Machine (Complex System)	~2.5 hr	~100 fg/ml
**abcam**	ELISA	Microplate Reader	~2.5 hr	~70 pg/ml
**This Work**	Colorimetric (Coffee-Ring)	Naked Eye	**~10 min**	**50 pg/ml** (Best LOD **3 pg/ml** for PSA, **30 times better than ELISA** [5])

Table.S2: Comparing state-of-the-art non-lateral flow point-of-care (POC) biosensors for SARS-CoV-2 detection, tested in the human samples. As the number of POC biosensors for detecting N protein (for a fair comparison) is very limited, we fixed the disease rather than the biomarker. Only one of the proposed methods provides better sensitivity than our Coffee-Ring biosensor, however, this method requires measurements instruments which prevents it to be adopted as a simple at-home diagnostic kit.

Article	Type	Method	Biomarker	Sample	Time	LOD
Raziq et al. [6]	Electrochemical	DPV[1]	N-Protein	Nasopharyngeal Swabs	15 min	~ 50 (pg/ml)
Vezza et al. [7]	Electrochemical	EIS[2]	S-Protein (RBD)	Saliva Samples	30 min	1.68 (ng/ml)
Soto and Orozco [8]	Electrochemical	EIS	S-Protein	Nasopharyngeal Swabs	15 min	18.2 (ng/ml)
Yakoh et al. [9]	Electrochemical	SWV[3]	IgM	Serum Samples	30 min	140 (pg/ml)
Funari et al. [10]	Optical (Spectroscopy)	LSPR[4]	S-Protein Antibody	Plasma Samples	30 min	80 (pg/ml)
Singh et al. [11]	Electrochemical	Glucometer + Magnetic Amplification	N-Protein	Oropharyngeal Swabs	60 min	250 (ng/ml)
Seo et al. [12]	Electrochemical	G-FET[5]	S-Protein	Nasopharyngeal Swabs	<1 min	100 (fg/ml)
**This Work**	**Optical (Colorimetric)**	**Naked Eye**	**N-Protein**	**Pulled Human Saliva Samples**	**~10 min**	**50 (pg/ml)**

[1] DPV: Differential Pulse Voltammetry

[2] EIS: Electrochemical Impedance Spectroscopy

[3] SWV: Square Wave Voltammetry

[4] LSPR: Localized Surface Plasmon Resonance

[5] G-FET: Graphene-Field Effect Transistor

Table.S3: Comparing state-of-the-art smartphone-based biosensors for POC protein detection. It should be noted that, due to the limited number of smartphone-based POCs, we included a diverse list of biomarkers for various diseases. As a result, comparing different methods solely based on their LODs is not meaningful.

Article	Type	Method	Biomarker	Time	LOD
Draz et al. [13]	Optical	Microfluidics + Nanoparticles	HBV[1], HCV[2], ZIKV[3]	50 min	250 (viruses/ml)
Boonkaew et al. [14]	Electrochemical	Microfluidics + NFC[4]	CRP[5]	15 min	7.6 (pg/ml)
Jankelow et al. [15]	Optical (RT-LAMP[6])	Microfluidics + RT-LAMP Setup	ZIKA RNA	17-32 min	2.70×10^5 (copies/ml)
Su et al. [16]	Electrochemical	Cyclic Voltammetry	CD63-Positive Exosomes	120 min	1.446 (µg/ml)
Tang et al. [17]	Optical (RT-LAMP)	Microfluidics + RT-LAMP Setup	COVID-19 RNA	45 min	5.0×10^3 (copies/ml)
Yuan et al. [18]	Magnetic	Magnetoelastic Immunosensing	CRP	38 min	235 (pg/ml)
Dutta et al. [19]	Optical (LSPR)	UV-Visible Spectroscopy	BSA Protein, Trypsin Enzyme	120 min	19.2 (µg/ml)
**This Work**	**Optical (Colorimetric)**	**Naked Eye**	**N-Protein, PSA[7], PCT[8], CEA[9]**	**~10 min**	**~3.0 (pg/ml)**

[1] HBV: Hepatitis B Virus
[2] HCV: Hepatitis C Virus
[3] ZIKV: Zika Virus
[4] NFC: Near-Field Communication
[5] CRP: C-Reactive Protein
[6] RT-LAMP: Reverse-Transcriptase Loop-Mediated Isothermal Amplification
[7] LSPR: Localized Surface Plasmon Resonance
[8] PSA: Prostate-Specific Antigen
[9] PCT: Procalcitonin
[10] CEA: Carcinoembryonic Antigen

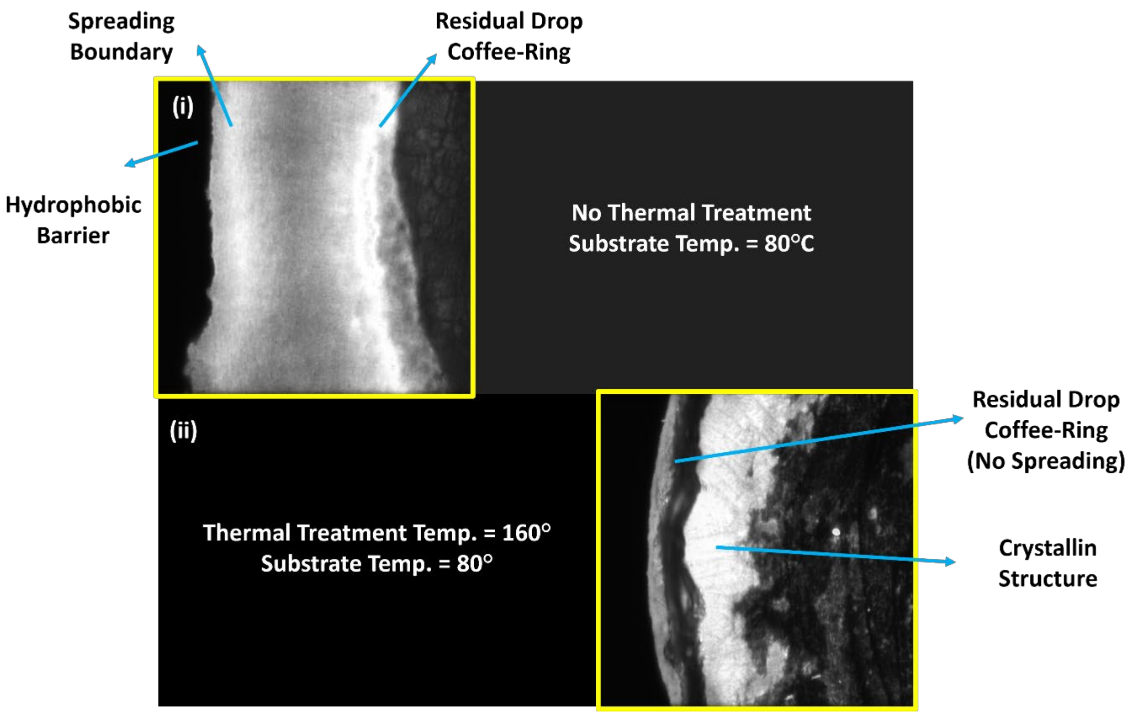

Figure.S1: Spreading induced particle flow. The droplet spreading through membrane pushes small molecules (e.g., salts) towards the hydrophobic barrier. Looking to two extremes, (i) no thermal treatment, and (ii) high temperature treatment, shows that in case (ii) all small molecules remain inside the residual drop, making crystallin objects at the coffee-ring, and preventing GNShs from getting access to biomarkers. Note, only images in yellow boxes are real, the rest is attached to represent relative position of the rings. In all cases, the evaporation has been done on a heated substrate. It should be noted as well that intensities are not comparable since they have been taken at different times and different conditions, which may cause variations in fluorescent dyes brightness.

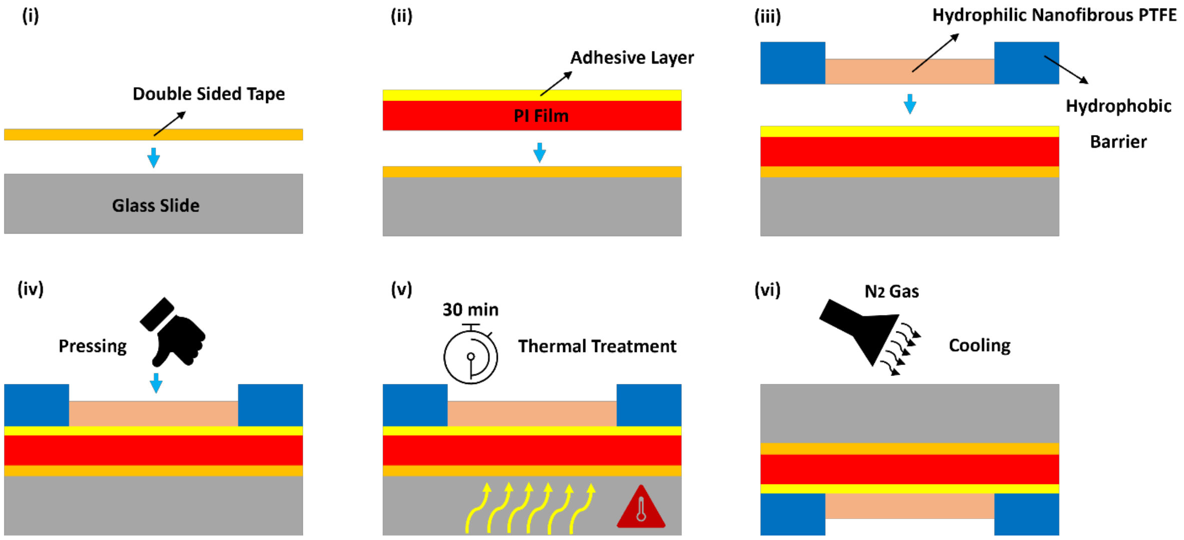

Figure.S2: Device fabrication process. (i) Attaching double-sided tape on top of glass slide. (ii) Fixing PI film with stacked adhesive layer to the double-sided tape-glass slide complex. (iii) Attaching the hydrophilic nanofibrous PTFE membrane on top of adhesive layer. (iv) Pressing membrane firmly to make sure having uniform attachment to the adhesive layer. (v) Placing device on hot plate at desired temperature for 30 min to perform thermal treatment. (vi) Lastly, cooling down the device using N_2 gas from backside of glass slide.

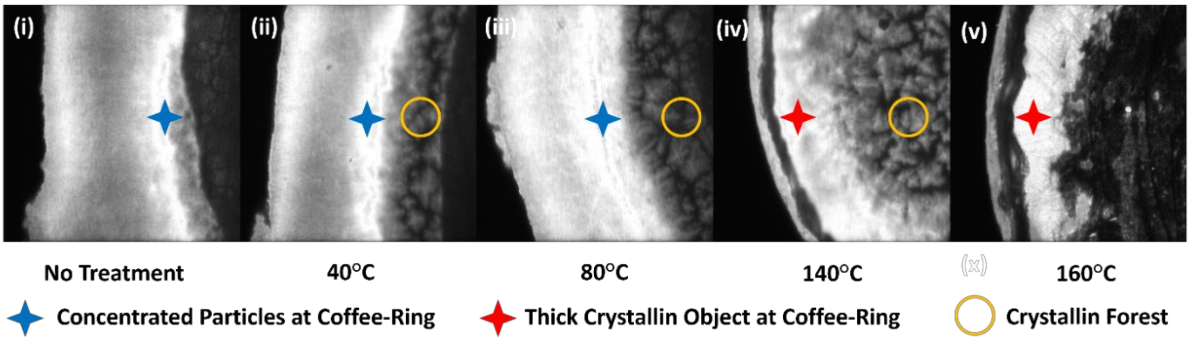

Figure.S3: Particle trapping in crystallin structures. Increasing thermal treatment temperature reduces droplet spreading through membrane by blocking the nanoholes within membrane. At lower temperatures, randomly distributed small crystals (crystallin forest) are formed inside the residual drop pattern (ii-iii), while at higher temperatures, the pattern changes to a combination of crystallin forest and thick crystalline object at the coffee-ring (iv), and single large crystallin object at the coffee-ring (v). Note, these crystalline objects prevent access to particles locating inside them. Note, intensities are not comparable since they have been taken at different times and different conditions, which may cause variations in fluorescent dyes brightness.

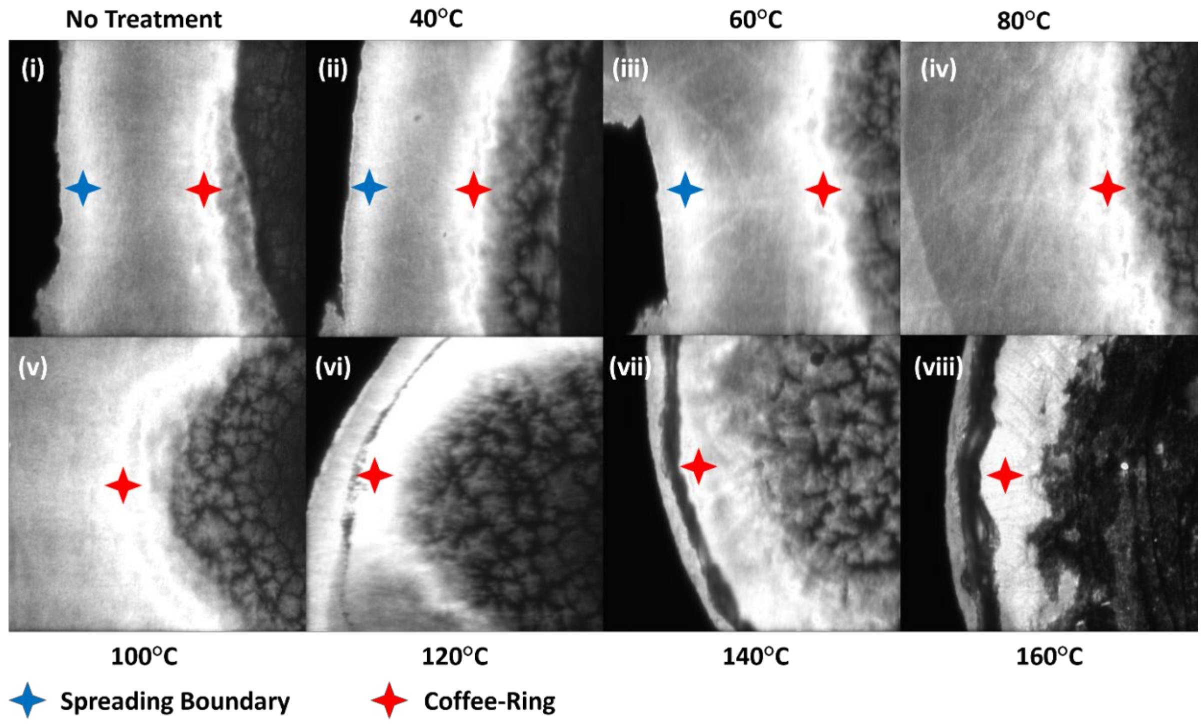

Figure.S4: Effect of thermal treatment temperature on the droplet spreading and the coffee-ring formation. Starting 120°C, there is no spreading anymore, causing thick crystallin object formation at the coffee-ring. Note, intensities are not comparable since they have been taken at different times and different conditions, which may cause variations in fluorescent dyes brightness.

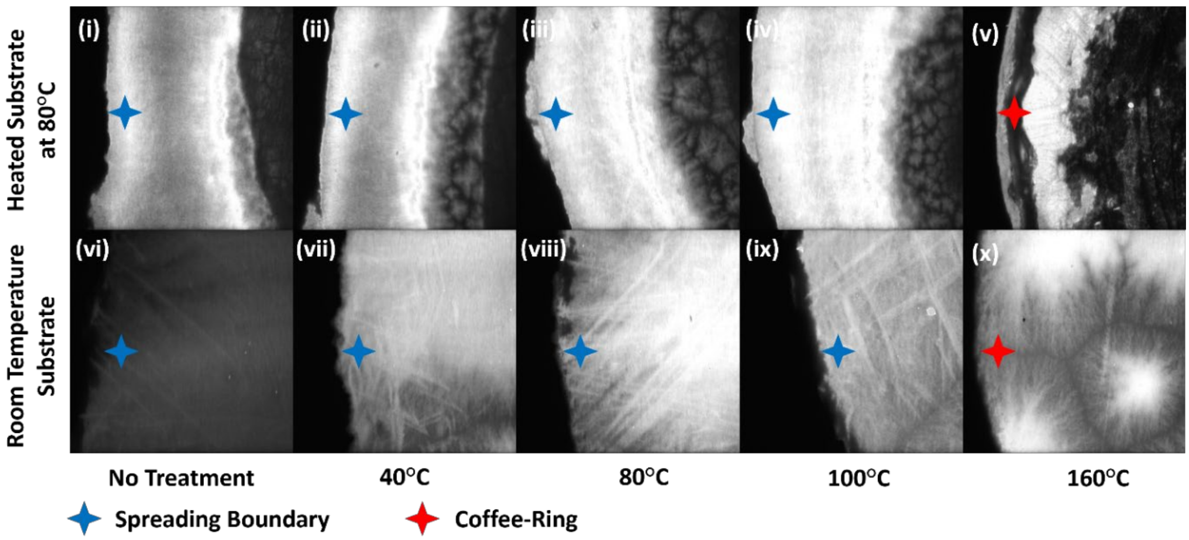

Figure.S5: Coffee-ring enhancement induced by heated substrate evaporation. Sample droplet evaporation at 80°C and room temperature (at different treatment temperatures) shows a significant enhancement in the coffee-ring intensity when droplet evaporates on a heated substrate, representing smaller timescales for evaporation induced flow towards boundaries than particle self-diffusion through residual drop. Note, intensities are not comparable since they have been taken at different times and different conditions, which may cause variations in fluorescent dyes brightness.

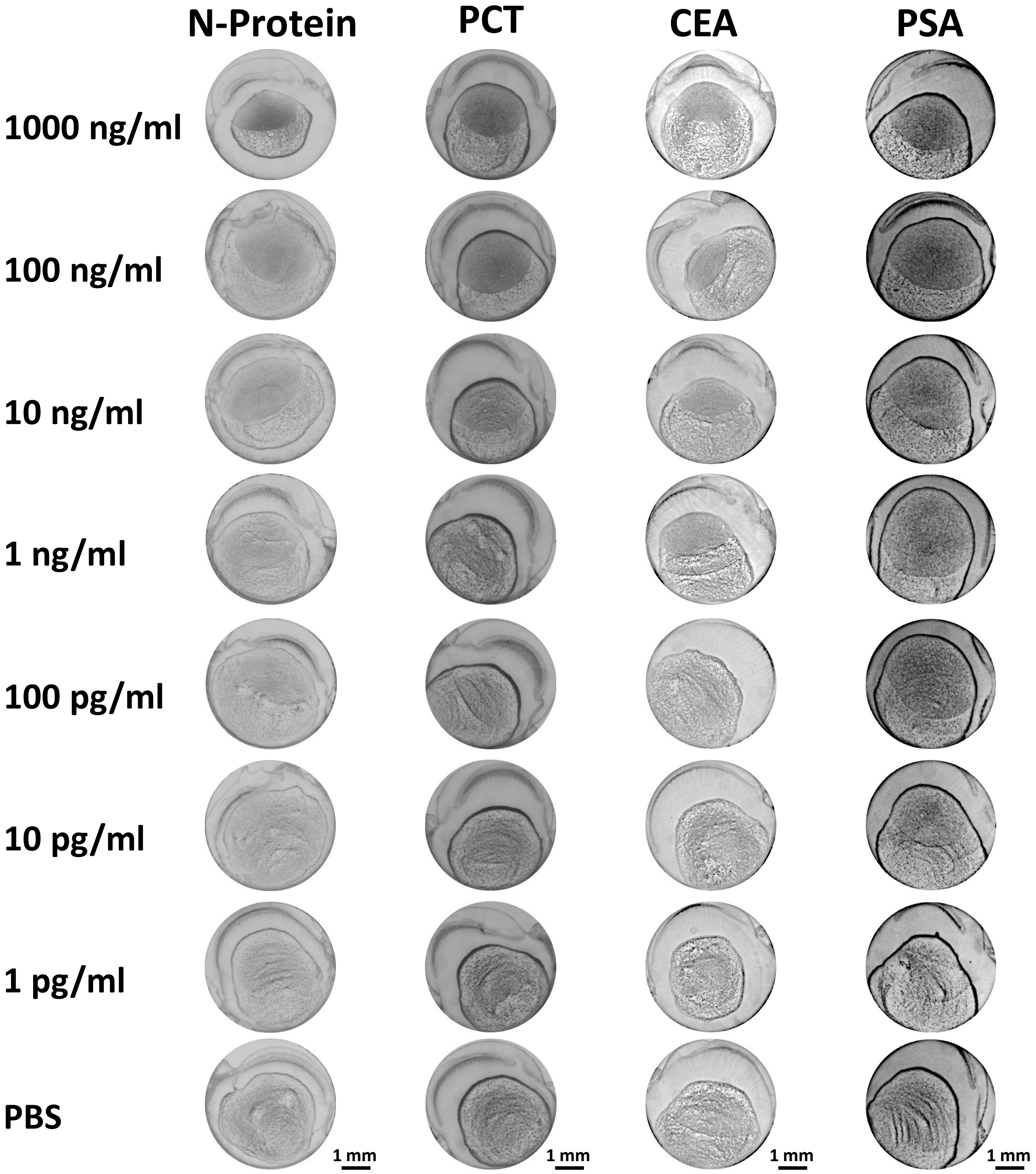

Figure.S6: Different biomarkers plasmonic asymmetric pattern at various concentrations. LOD for each case can be found in Figure.6e.

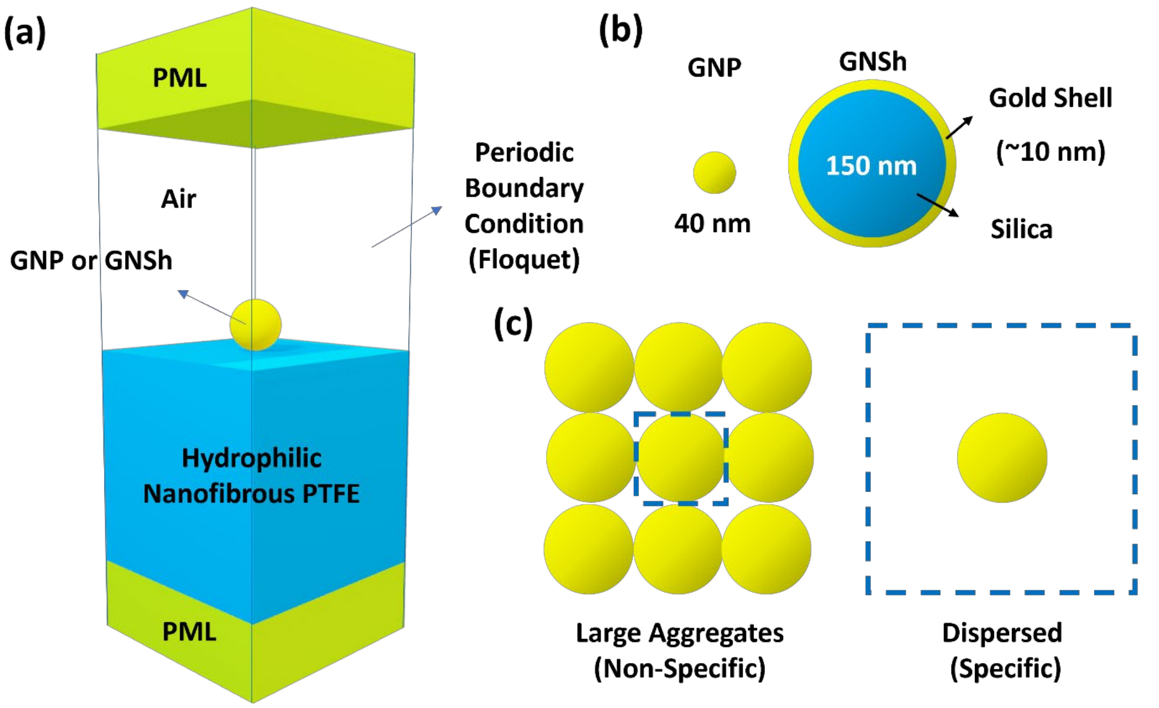

Figure.S7: Numerical modeling setup. (a) Schematics of the simulation box. We applied periodic boundary conditions on all side walls. (b) Plasmonic particle structure. GNP is modeled by placing a 40 nm sphere on the membrane using Drude-Lorentz model and GNSh is modeled with a 150 nm silica sphere covered by a 10 nm of gold (Drude-Lorentz). (c) To simplify the numerical simulation of specific and non-specific aggregates, we manipulated particle-to-particle distance, assuming highly concentrated as non-specific vs. dispersed distribution as specific.

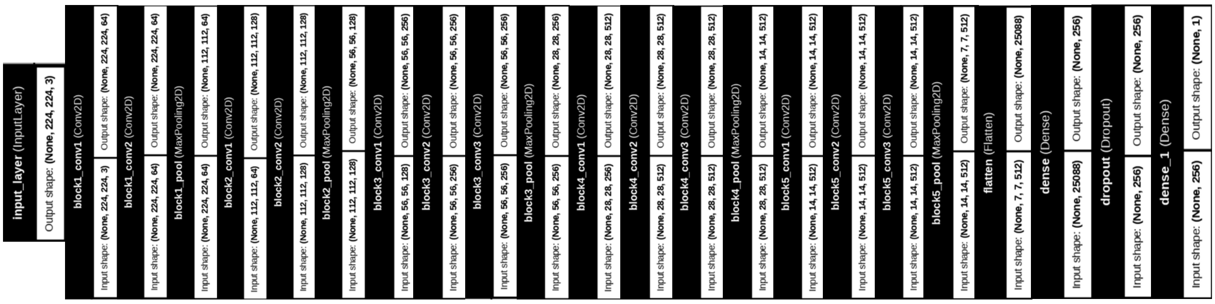

Figure.S8: CNN network with VGG-16 architecture for positive vs. negative classification. Additional information is provided in [20].

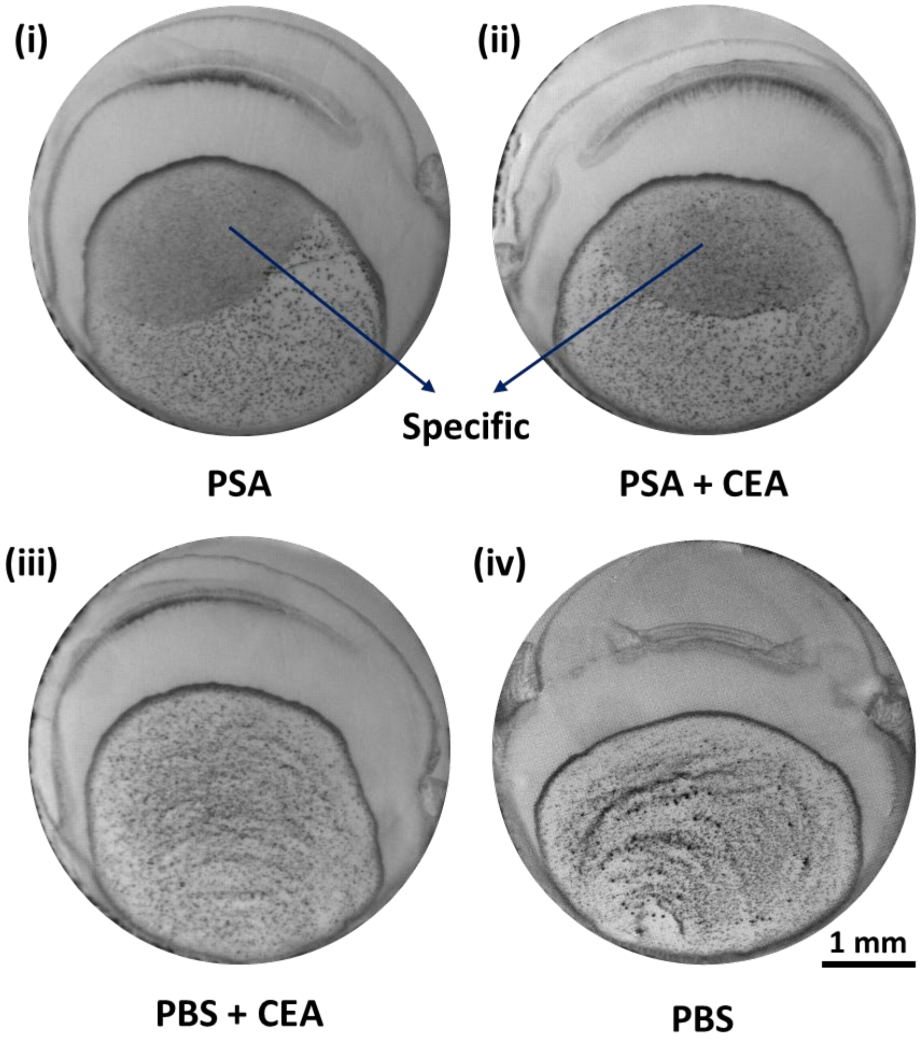

Figure.S9: Specificity test. (i) PSA only sample at 1000 ng/ml, (ii) mixture of PSA and CEA at 1000 ng/ml, (iii) PBS (buffer) and CEA at 1000 ng/ml, and (iv) PBS only sample. Specific pattern is visible only in the PSA spiked samples. Note that we used PSA antibody coated GNShs.

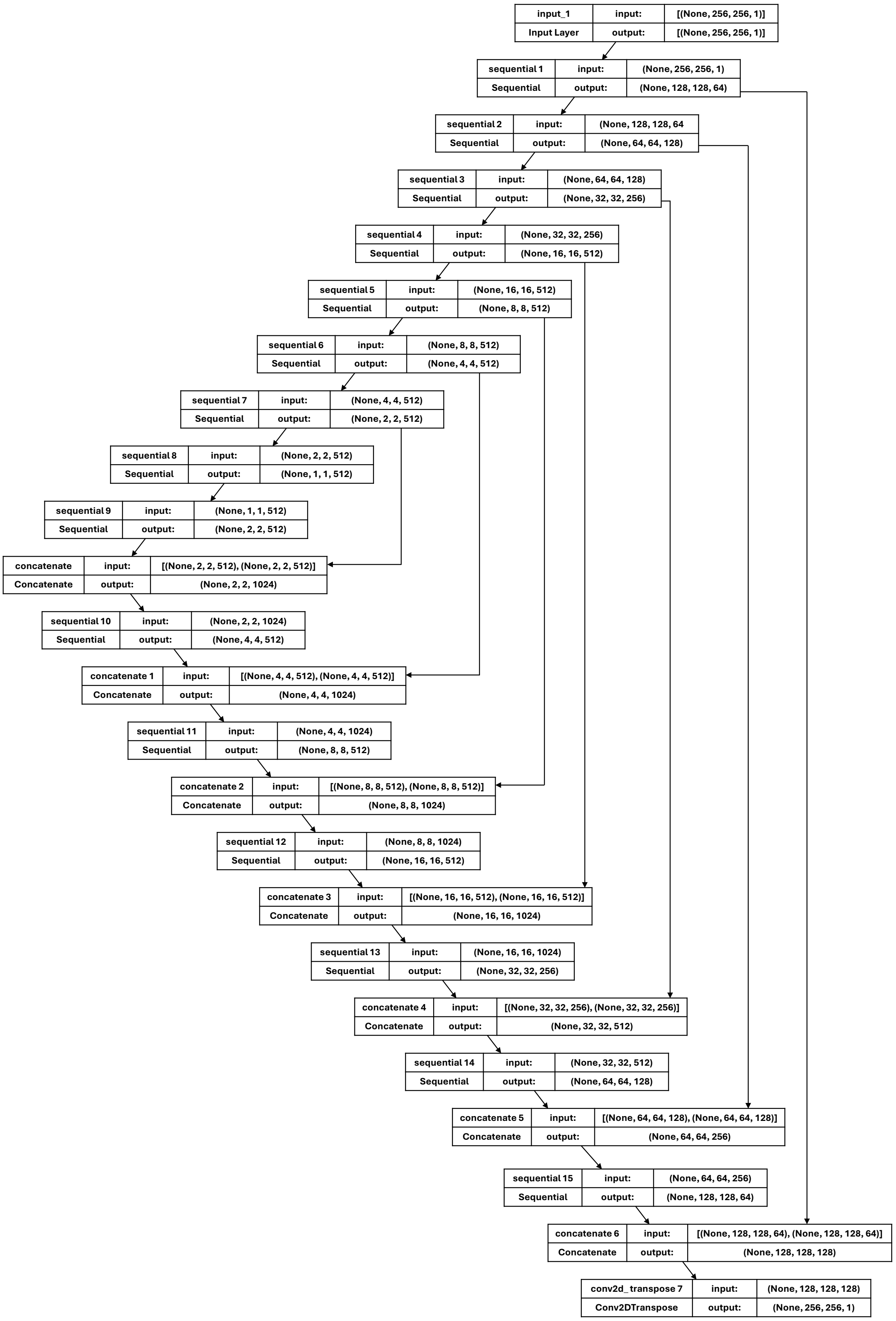

Figure.S10: Generative network architecture based on the U-Net structure for segmentation [21].

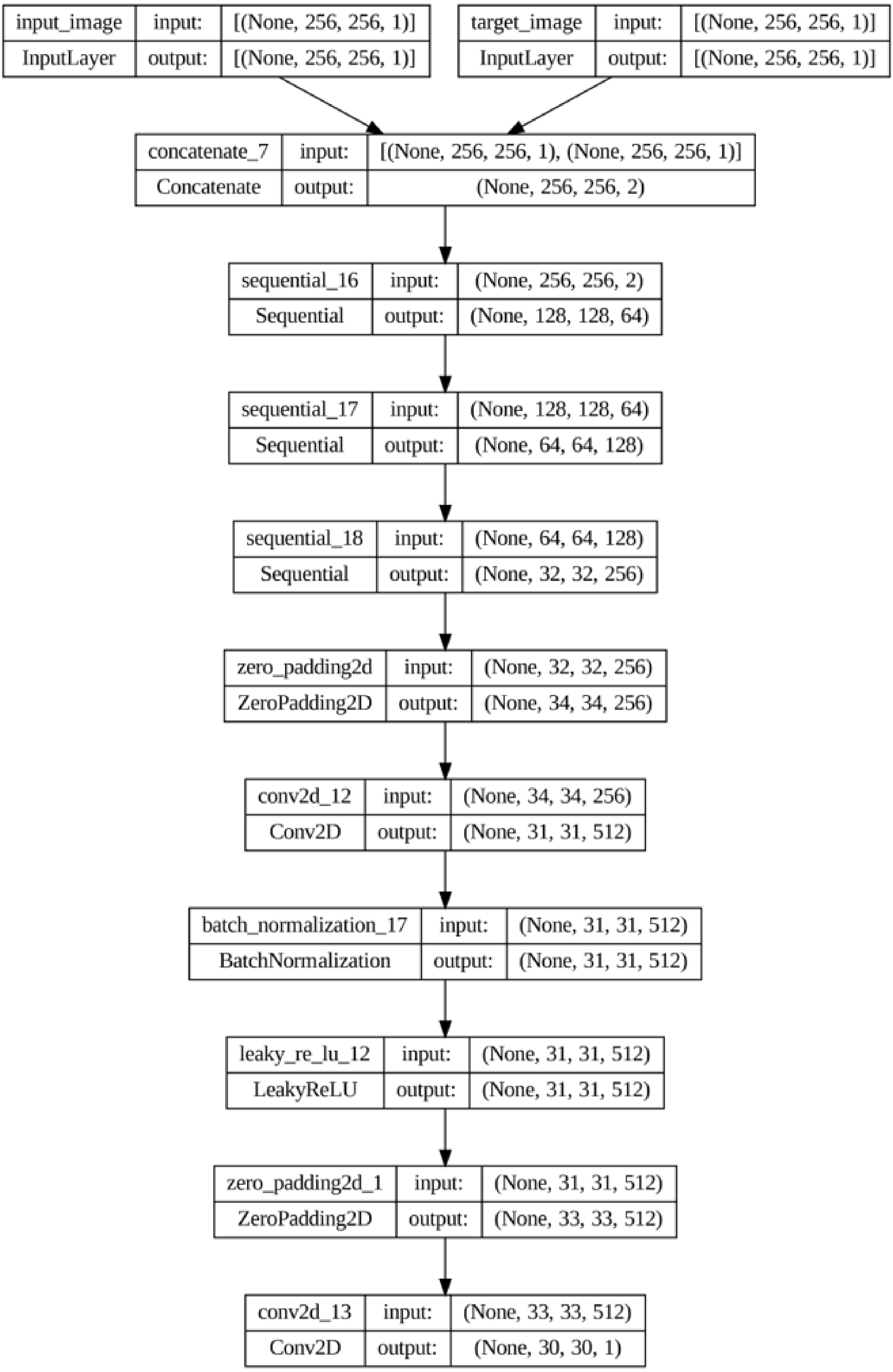

Figure.S11: Discriminator network. The design is based on the CNN architecture.

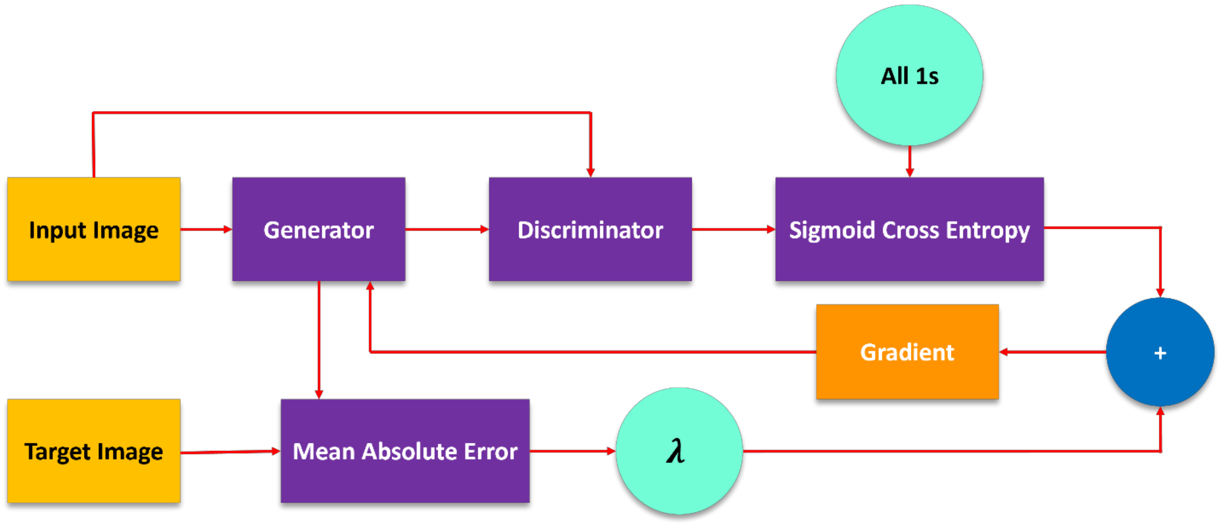

Figure.S12: Training process for generative network. λ controls the level of generativity of the network, we set it at 100 based on the original Pix2Pix method [22].

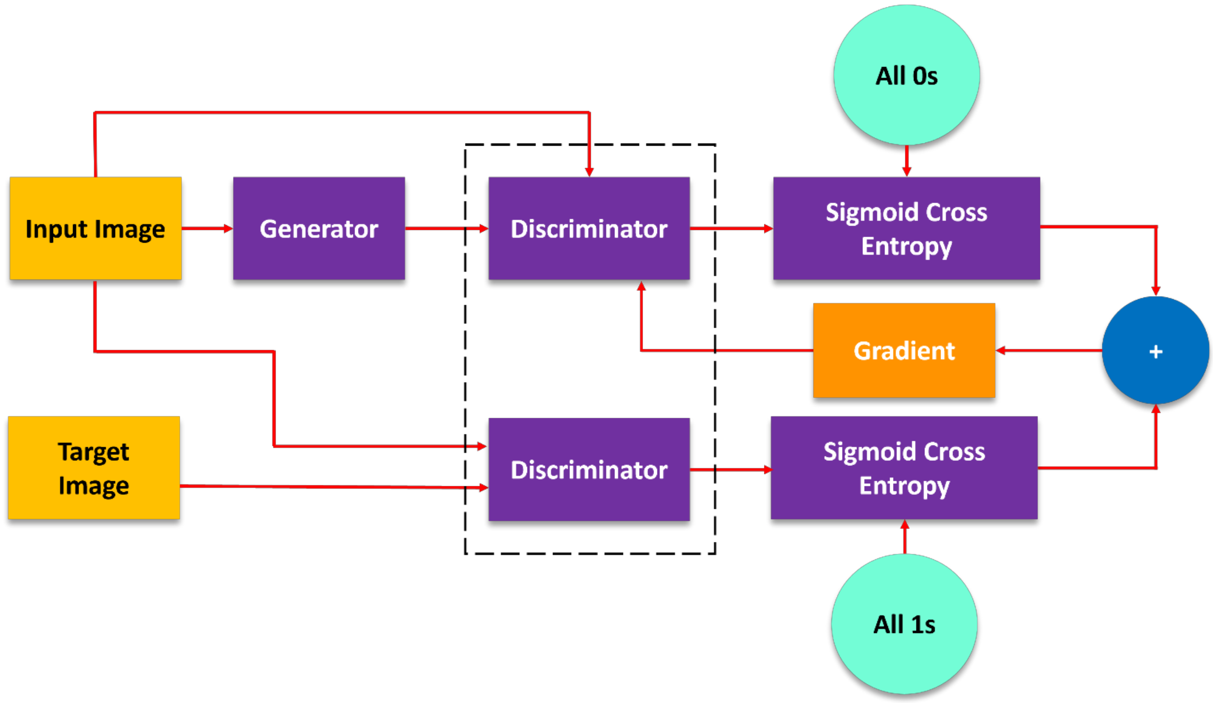

Figure.S13: Training process for the discriminator network. Note, generative and discriminator networks are trained together. Based on Pix2Pix method [22].

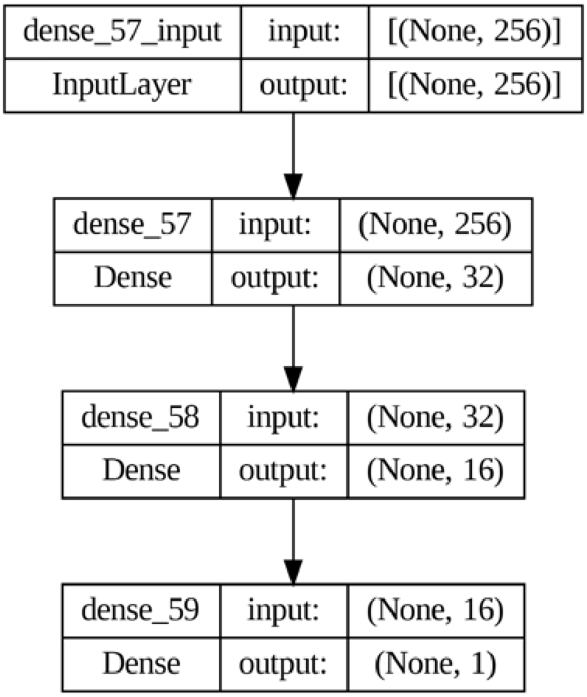

Figure.S14: FC network architecture for concentration quantification from coffee-ring centerlines.

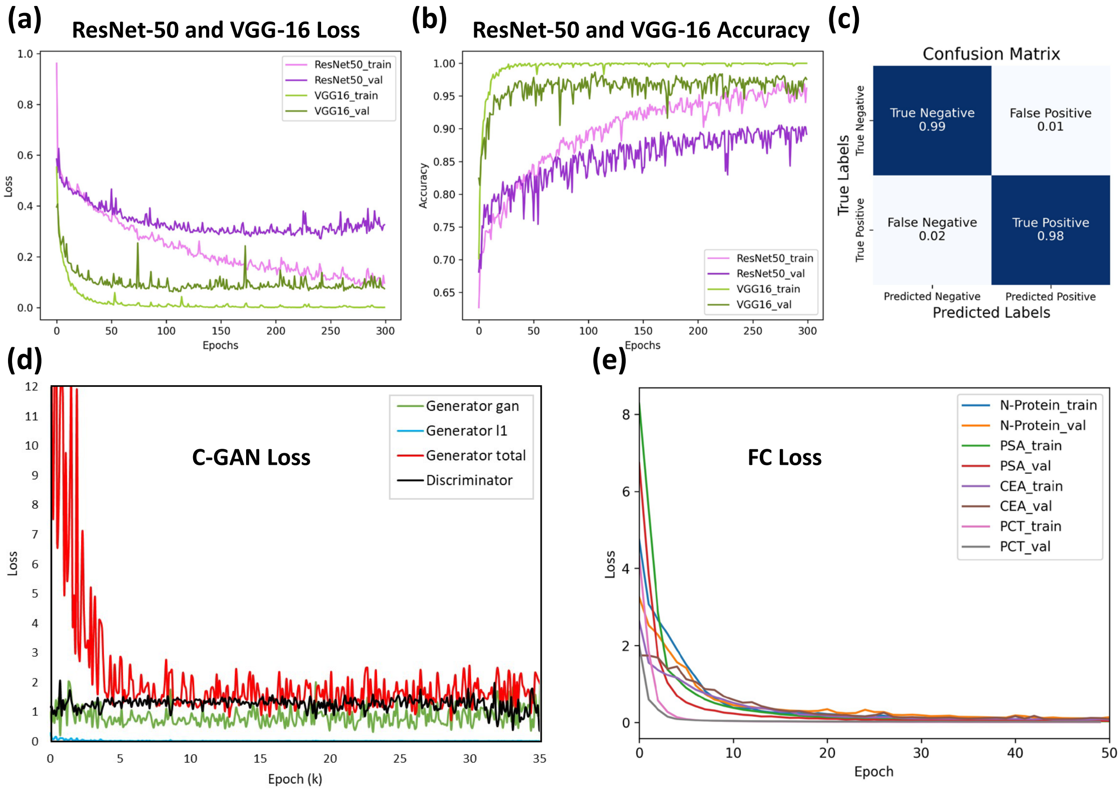

Figure.S15: Training metrics. (a-b) The loss and accuracy curves for VGG-16 and ResNet-50 during both training and validation, highlighting the superior performance of VGG-16. (c) The confusion matrix for VGG-16 model

predictions on the validation set. We conducted a similar analysis as presented in [23] (d) For the C-GAN model, the generator and discriminator losses exhibited the expected oscillatory behavior, indicating that the optimal stopping point is best determined by comparing generated images with manually segmented labels. To do so, images were plotted every 1,000 epochs. Additional details on the stopping criteria for C-GAN training can be found in [24], [25]. (e) Loss variations during the training and validation of the FC network for all biomarkers tested, showing rapid model convergence. Our results indicate that the basic FC network is adequate for analyzing coffee-ring crossline data and quantifying concentrations.

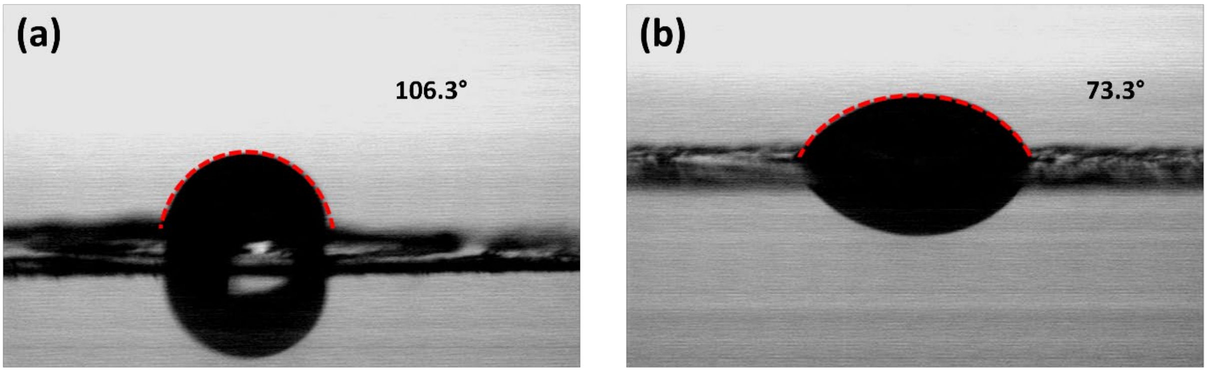

Sample Drop on Thermally Treated Hydrophilic Nanofibrous PTFE (at 80°C)

GNShs Drop on Thermally Treated Hydrophilic Nanofibrous PTFE (at 80°C)

Figure.S16: Contact angle measurements using sessile drop method. Same thermally treated substrate shows hydrophobicity to the sample drop, while hydrophilicity to GNSh drop, due to the different buffer solution used for each of these droplets (see, Materials and Methods). This difference causes sample droplet to have higher coffee-ring enhancement, suitable for the biomarkers' enrichment, and less coffee-ring effect for the plasmonic droplet, leading to less GNShs crowding at the detection zone, eventually making a clear pattern.

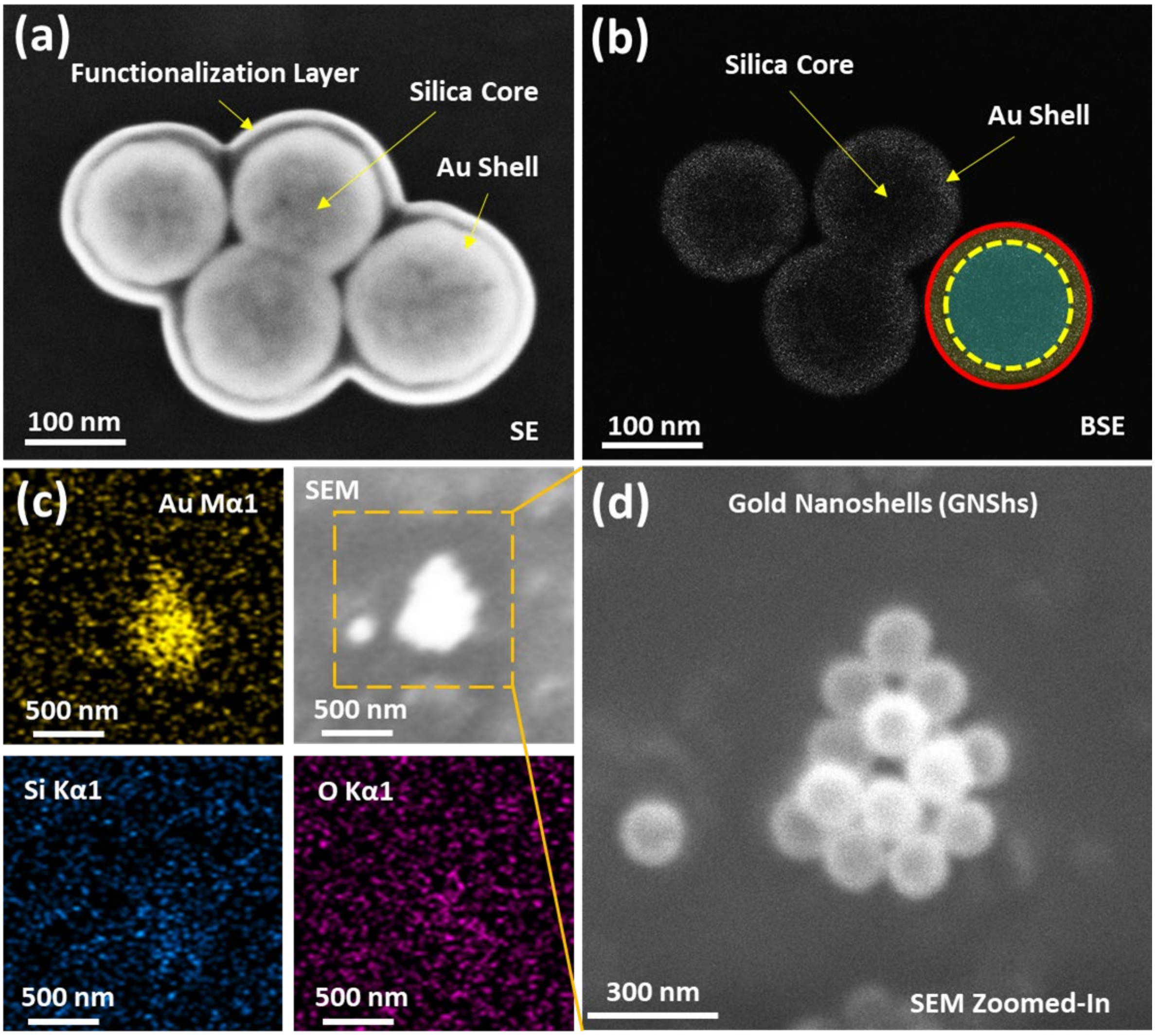

Figure S17: Structural analysis of gold nanoshells (GNShs). (a) Scanning electron microscopy (SEM) image of GNShs captured in the secondary electron (SE) mode, where scattered electrons in the forward direction provide a clear view of the overall morphology. The ~20 nm-think gold shell, ~110 nm in diameter silica core, and the outer functionalization layer are distinguishable. (b) SEM image of the same GNShs cluster acquired in the backscattered electron (BSE) mode. The BSE image offers a clear visualization of the internal structure, where heavier gold atoms (Au) backscatter more electrons with higher contrast as compared with that of the silica core. The carboxyl chains in the functionalization layer are not visible in the BSE mode due to their low molecular weight and less dense packing. (c) Energy dispersive X-ray spectroscopy (EDS) data collected from the GNShs cluster. The elemental compositions primarily with Au, Si, and O, are captured to show the strong Au signal in comparison to those of Si and O. However, due to the resolution limitation in the EDS mode, the resulting image of the cluster has relatively low spatial resolution. By correlating the high-resolution SEM images (a-b) with the EDS data, the shape, size, and elemental composition of GNShs can be analyzed. (d) High-resolution SEM image of the GNShs cluster used for EDS analysis. The analysis is consistent with previously reported nanoshell studies [26], [27].

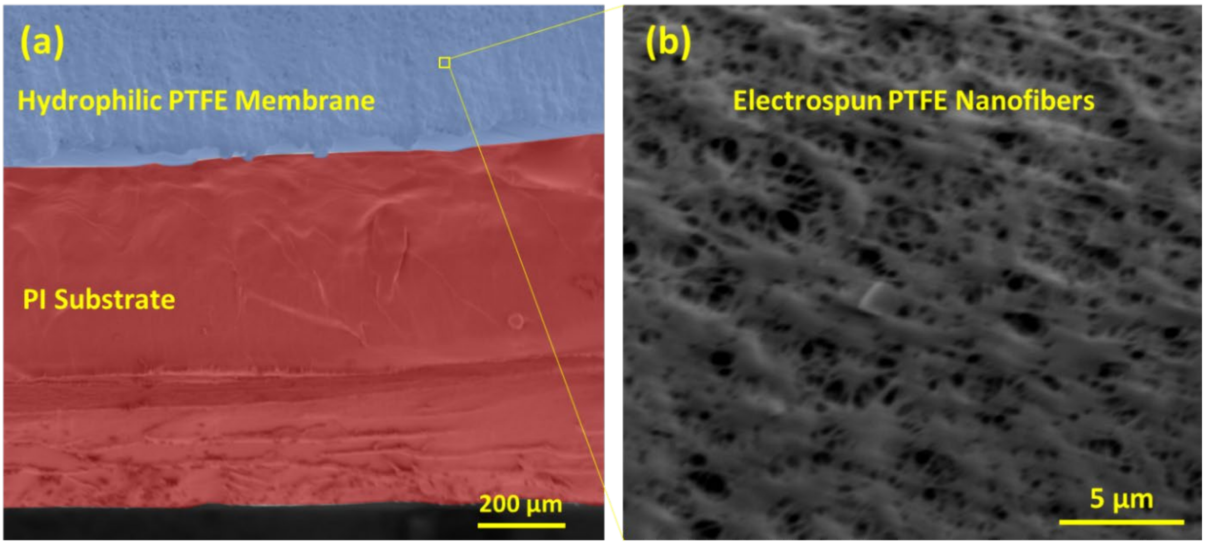

Figure.S18: (a) SEM image of the hydrophilic nanofibrous PTFE membrane (blue) attached on the PI film (red). (b) Zoomed-in view of the electrospun PTFE nanofibers. Note that the sample was coated with gold as a conductive layer to enhance the SEM image. The sample has been attached to the SEM sample holder at 45°. The measured average thicknesses for PI film and the PTFE membrane are 110 µm and 6 µm, respectively.

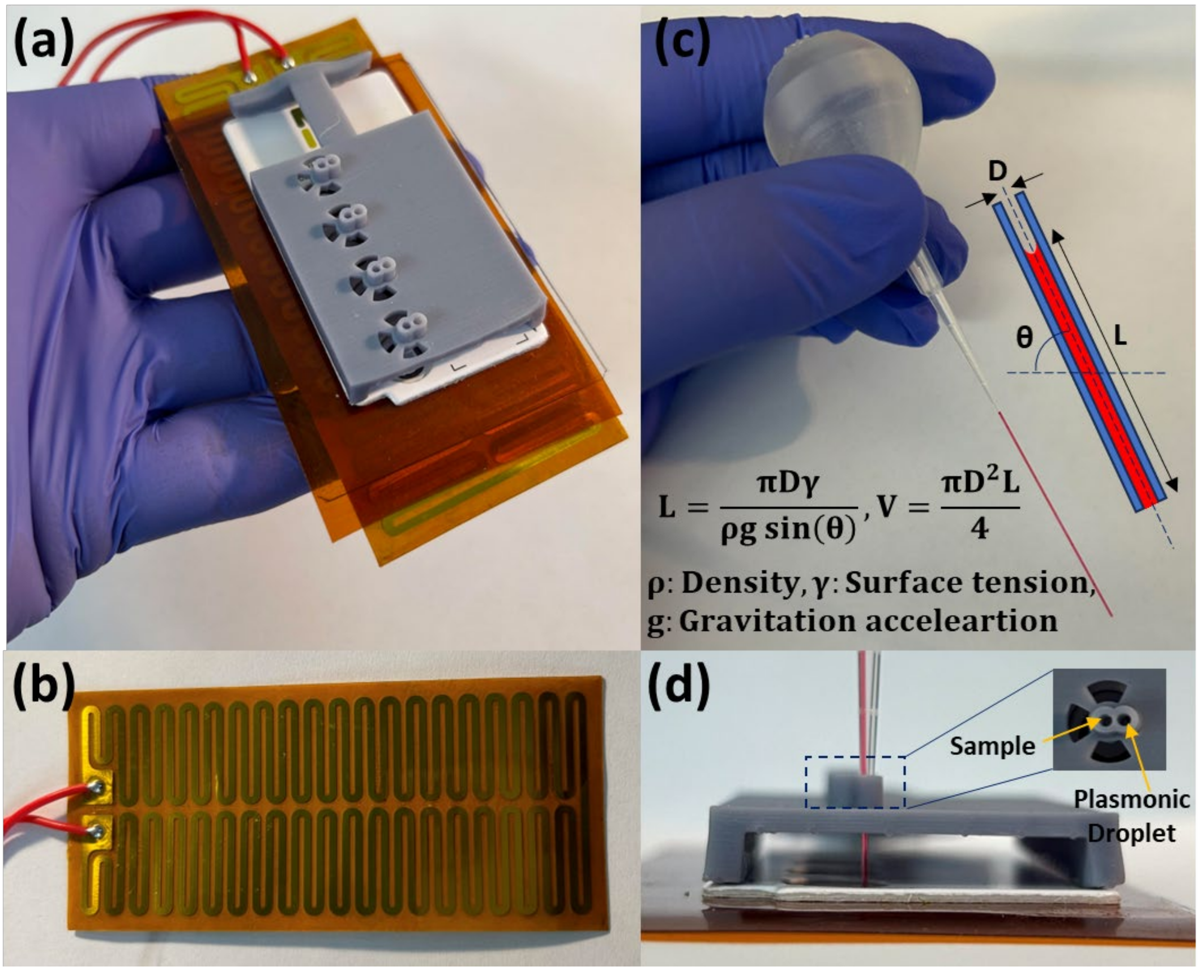

Figure.S19: (a) At-home multiplex coffee-ring biosensor kit. (b) A film heater made of patterned copper wires on a PI substrate, designed to provide 80°C at 12 V applied voltage. (c) A glass microtube to facilitate handling of the sample and plasmonic solution at microliter volumes. The tubes were cut into estimated lengths for the sample and plasmonic drops, 5 µl and 2 µl, respectively, providing accurate yet simple control over the required volumes. Based on the provided equation, the user can tilt the microtube to fill the entire tube. A 3D-printed flexible bulb attached to a pipette tip was used to hold the microtube, enabling a simple droplet release mechanism. (d) A 3D-printed mount for precise control over the placement of the droplets, ensuring a consistent gap between them for higher repeatability, while also facilitating the droplet release mechanism. Open windows were designed to provide alignment markers for accurate positioning of the mount relative to the substrate.

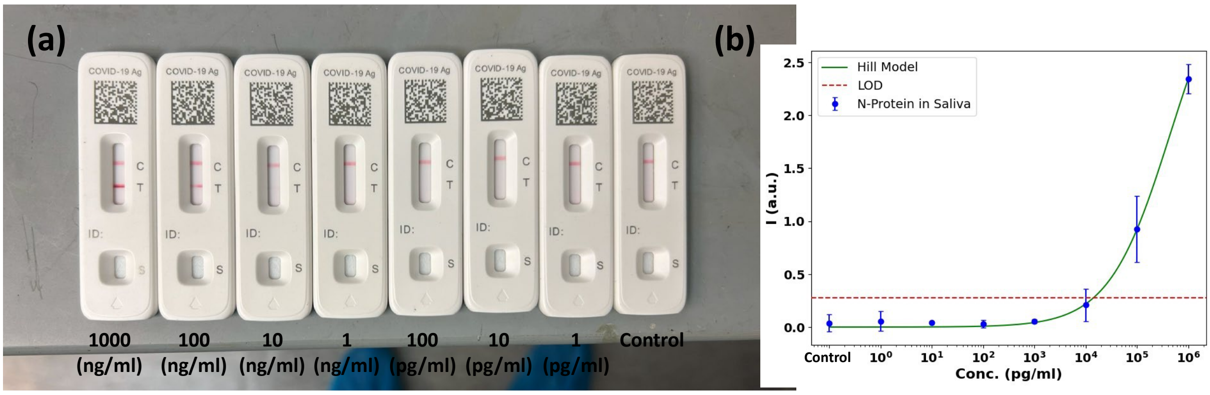

Figure.S20: LFIA tested with N-protein of SARS-CoV-2 containing human saliva. (a) Images of LFIAs tested with the equivalent volume to the coffee-ring biosensor under varying concentrations from 1 pg/ml to 1000 ng/ml. (b) Measured responses of LFIA, as explained in Materials and Methods, showing an estimated LOD of approximately 10 ng/ml. It should be noted that coffee-ring LOD in human saliva (100 pg/ml) is more than two orders of magnitude better than the equivalent-volume case. Note, values are expressed as mean ± standard deviation, based on n = 3 sample lines.

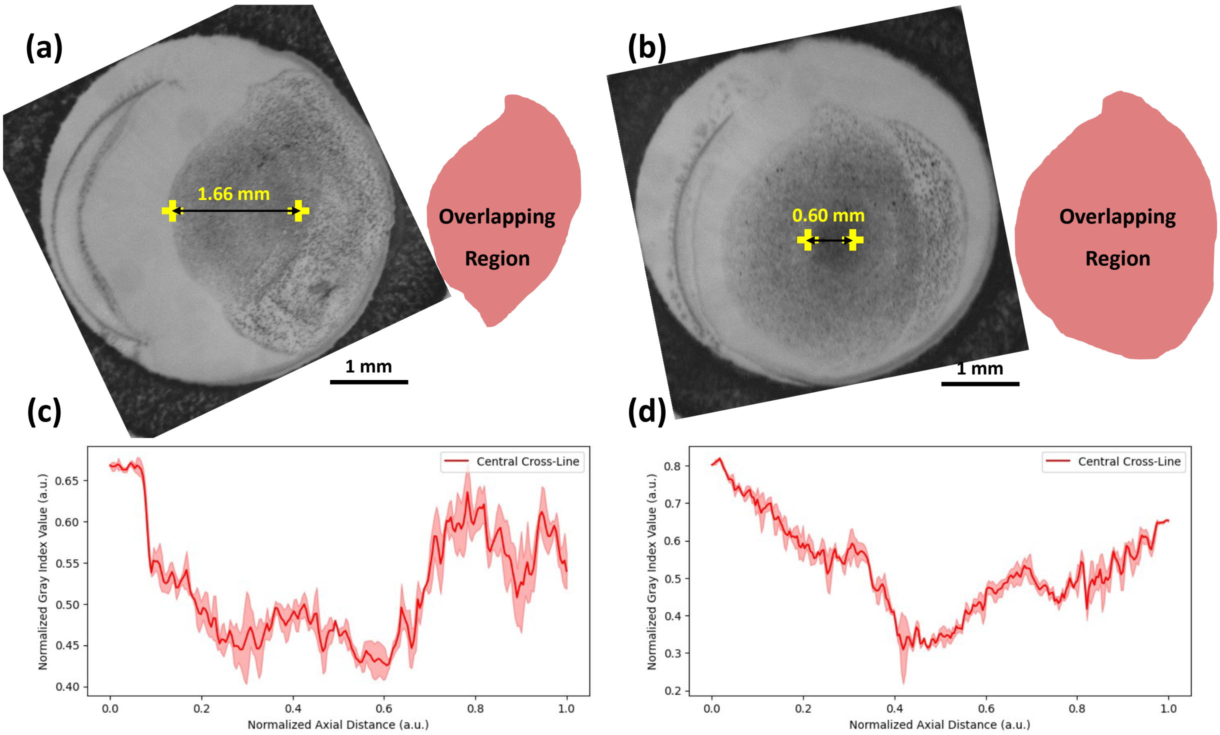

Figure.S21: Effect of the droplet gap distance. (a-b) Asymmetric coffee-ring patterns for the N-protein of SARS-CoV-2 at a 100 ng/ml concentration with gap distances of 1.66 mm and 0.6 mm, respectively. The red regions indicate the overlapping area between the sample and plasmonic droplets. (c-d) Central crossline gray index patterns for the 1.66 mm and 0.6 mm gap distances, respectively, showing distinct characteristics due to the variation in gap distance. The variations in the crossline patterns highlight the importance of maintaining consistent droplet placement locations and gap distances for repeatable results. Data are expressed as mean values ± standard deviation, based on n = 3 sample lines.

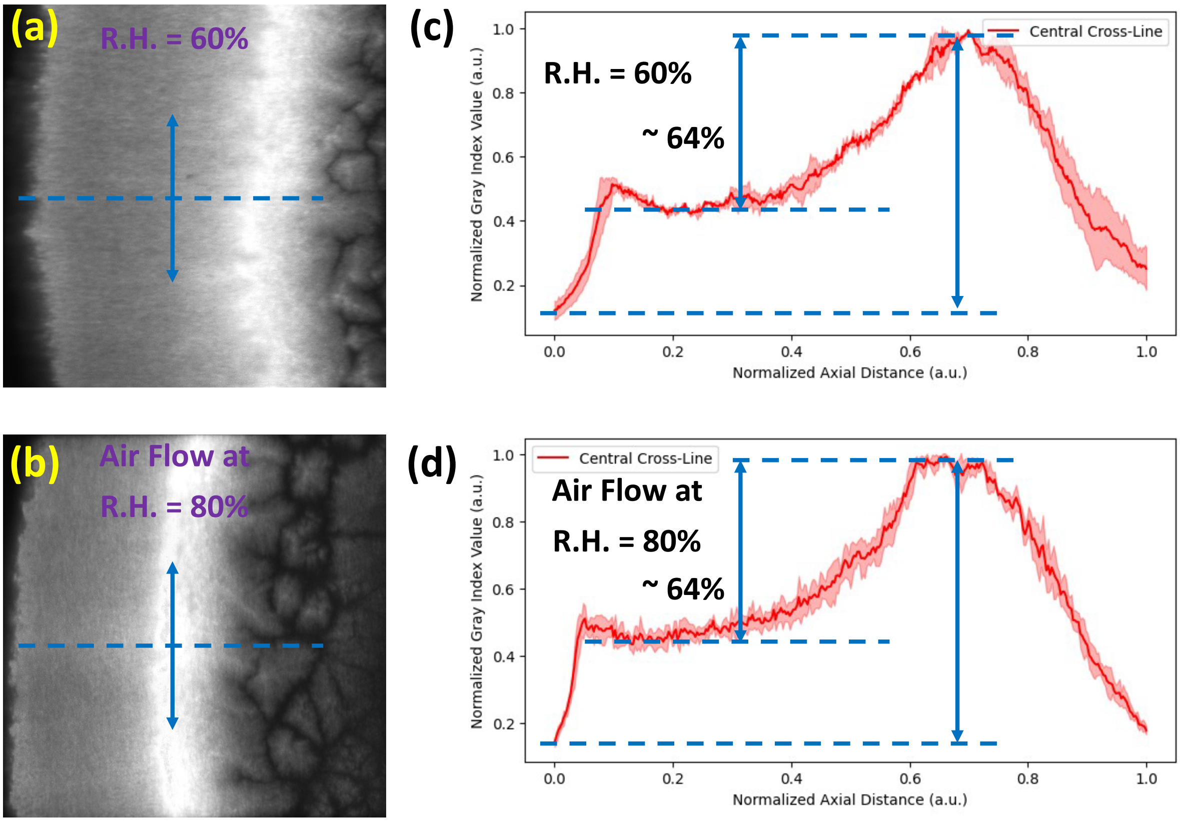

Figure.S22: Effect of humidity on the coffee-ring under humid air flow. (a-b) Fluorescence images of the deposited pattern created by fluorescent dyes at a 1 µg/ml concentration under room humidity (~60% relative humidity, R.H.) and cyclic humid air flow (~80% R.H., 2s off, 4s on), respectively. The substrate was heated to 80°C, and thermal processing lasted only 1 minute to ensure all droplets could wet the entire membrane, making the comparison more consistent. (c-d) Average crossline gray index intensities over multiple sections show similar coffee-ring patterns, with a 64% increase in intensity compared to the background for the room humidity and the humid air flow cases, respectively, indicating that humidity has a negligible effect on the performance of the coffee-ring biosensor. The relative position of the coffee-ring line varies slightly due to small differences in droplet placement. It should be noted that humidity can significantly alter the coffee-ring pattern in non-heated droplet evaporation on non-porous substrates, especially for droplet sizes under 100 µm [28]. However, in our case, substrate porosity and elevated temperature dominate the pattern formation. Additionally, evaporation time and the size of the residual droplet, along with the resulting crystallin pattern within the residual droplet, can vary in each case. Note, data are shown as mean values ± standard deviation, based on n = 3 sample lines.

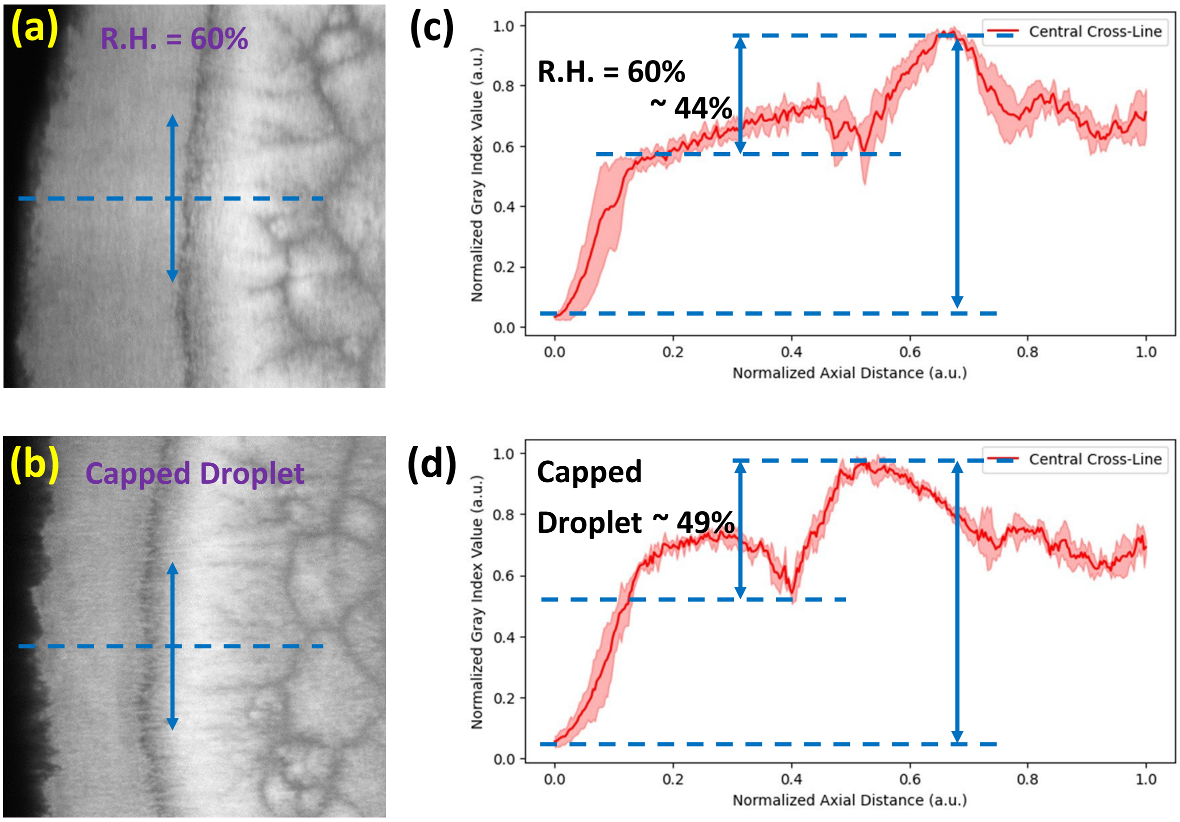

Figure.S23: Effect of humidity on the coffee-ring under capped condition. (a-b) Fluorescence images show the patterns formed by fluorescent dyes at a concentration of 10 μg/ml under room humidity (~60% R.H.) and inside a capped environment (with ~3 mm space around the droplet), respectively. The substrate was heated to 80°C, and thermal treatment lasted just 1 minute to ensure complete membrane wetting by all droplets, ensuring a fair comparison. (c-d) Average crossline gray index intensities over several sections indicate that the coffee-ring patterns are approximately similar, with a 44% and 49% intensity increase relative to the background for the room humidity and capped droplet conditions, respectively. This suggests that humidity has a very limited impact on the coffee-ring biosensor's performance, even under extreme conditions such as capping the droplet. Slight variations in the coffee-ring's position are due to minor differences in droplet placement. It is worth noting that, in non-heated droplet evaporation on non-porous substrates, humidity can greatly affect coffee-ring formation, particularly for droplets smaller than 100 μm [28]. However, in this case, the porous substrate and elevated temperature are the primary factors affecting the pattern formation. Furthermore, the evaporation time and the final droplet size, along with the crystalline pattern formed in the residual droplet, can differ in each scenario. In contrast to humid air flow experiments (see **Figure S22**), the humidity effect is slightly higher here, as the capping process can increase localized humidity to nearly 100% R.H.. Additionally, the higher concentration of fluorescent dyes (10x higher) leads to larger crystalline patterns inside the residual droplet. Values are represented as mean ± standard deviation, based on n = 3 sample lines.

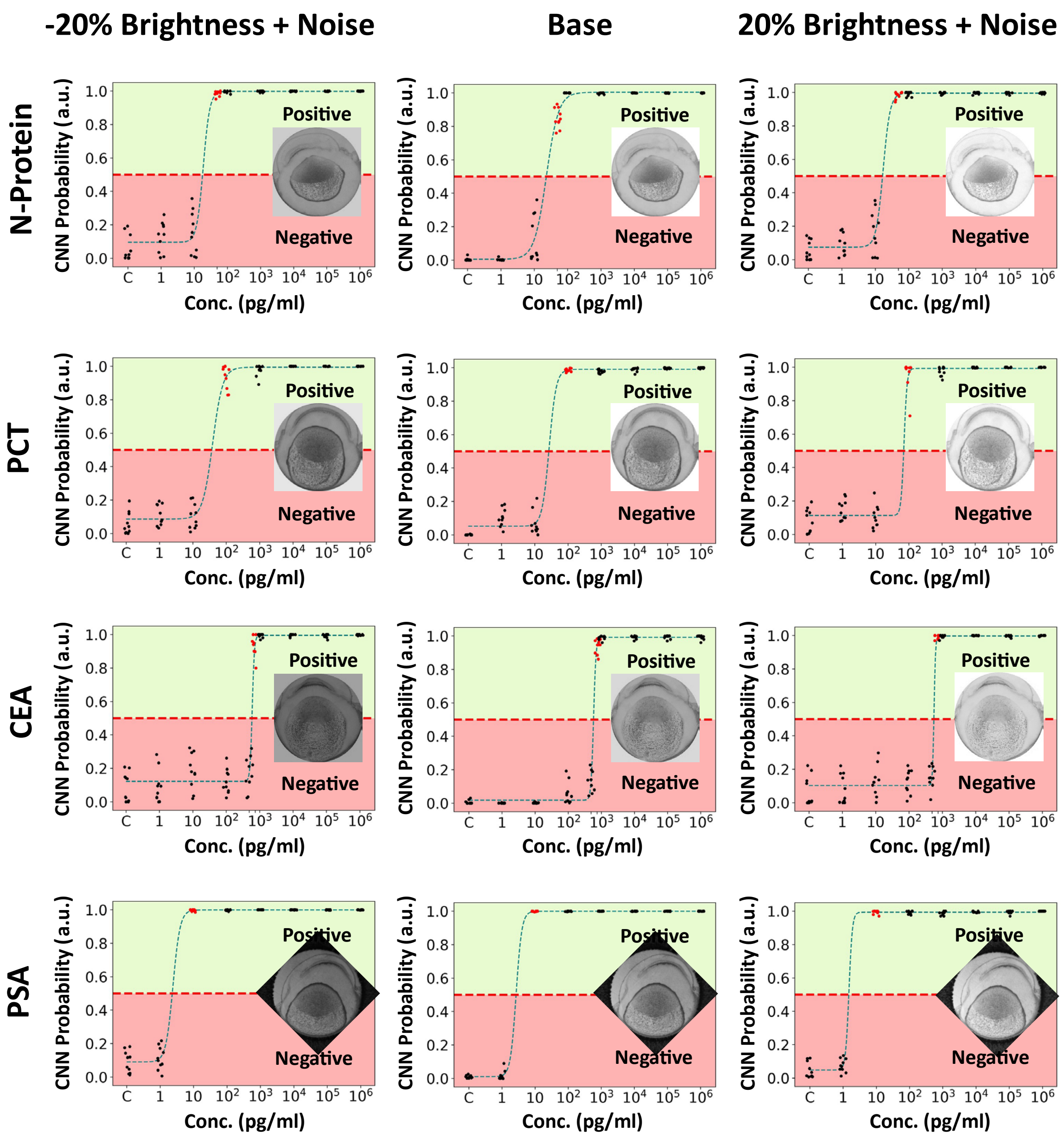

Figure.S24: Effect of brightness and noise variations on CNN network for the sample screening. To model the influence of varying lighting conditions and sensor noise on the neural network, we introduced virtual brightness changes and added random noise to the test dataset, subsequently generating new predicted probabilities using the VGG-16 model. The results exhibited minimal variation, further demonstrating the robustness of the trained model. Insets show an image of the plasmonic pattern under these conditions, derived from a 1000 ng/ml concentration for better visualization.

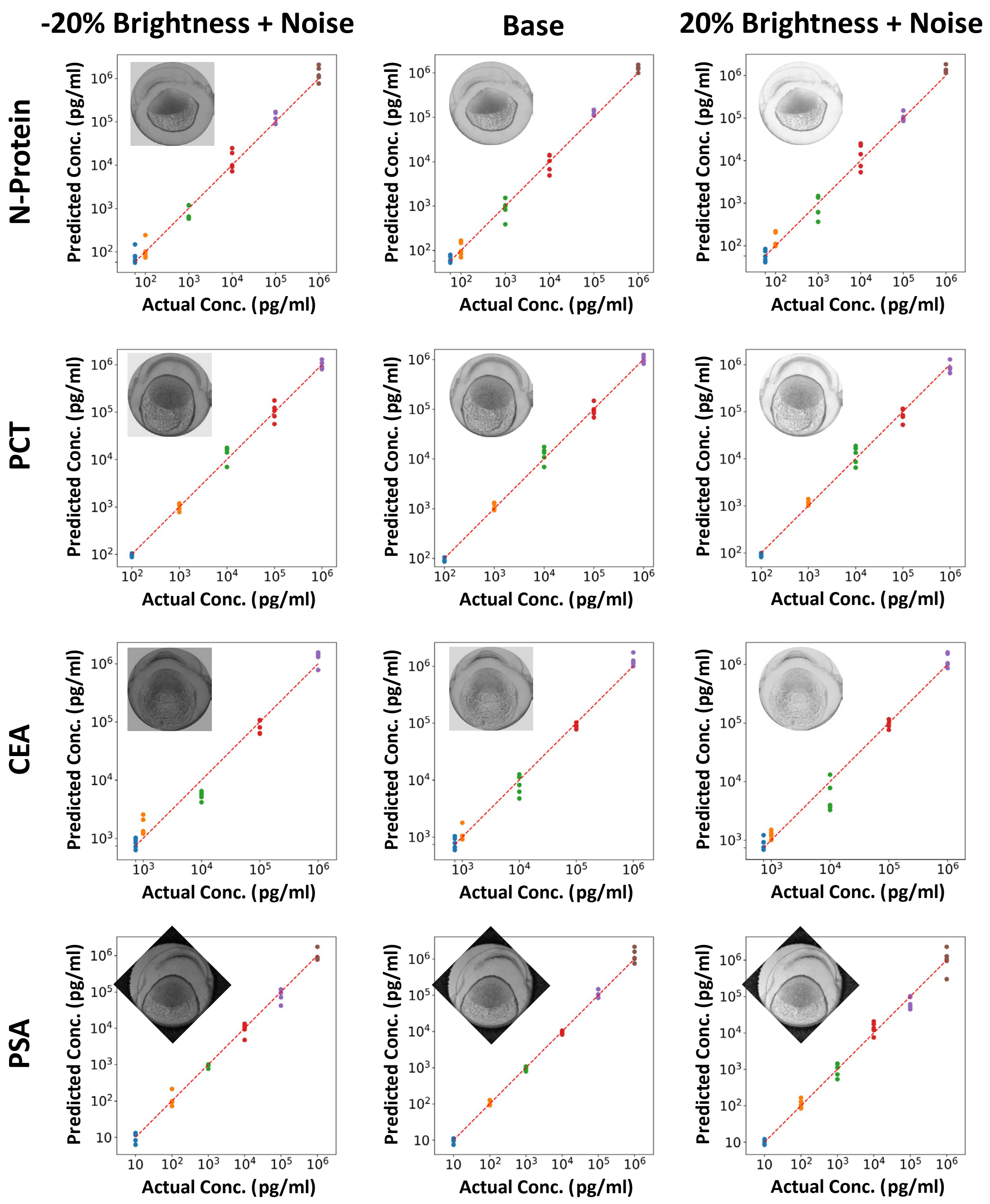

Figure.S25: Effect of brightness and noise variations on FC network for the concentration quantification. To simulate the impact of different lighting conditions and sensor noise on the neural network model, we applied virtual brightness adjustments and introduced random noise to the test dataset, then obtained the newly predicted concentrations from the model. The results show minimal variation, further proving the robustness of the trained model. It is important to note that the model's self-calibration, achieved by subtracting the background gray index value, makes it naturally resilient to brightness changes, as long as data is not lost due to saturation at very high brightness or masked by noise at very low brightness. Insets display an image of the plasmonic pattern under the respective conditions, taken from a 1000 ng/ml concentration for clarity.

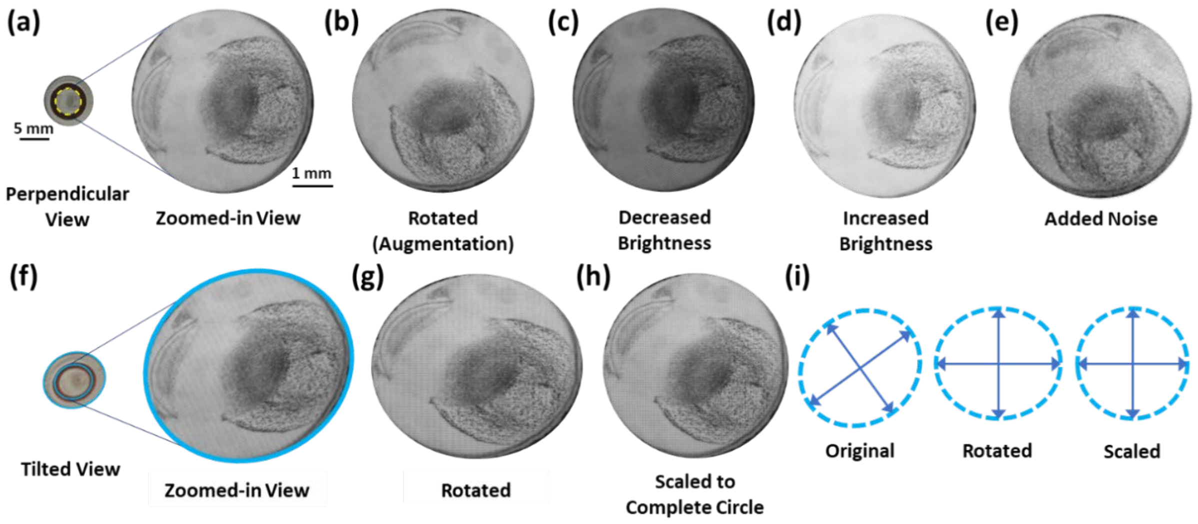

Figure.S26: Image processing on the coffee-ring biosensor outputs. (a) A smartphone image captured perpendicularly, showing the asymmetric plasmonic pattern of N-Protein at a concentration of 10 ng/ml, with a zoomed-in view on the right. (b-e) Image processing steps applied to the asymmetric plasmonic pattern, including rotation (used for data augmentation), brightness adjustment, and noise addition. It is important to note that while only rotation was used for augmentation, the trained model exhibited excellent robustness against variations in lighting and noise. (f) A smartphone image of the same sample captured at a tilted angle, with a zoomed-in view on the right. (g-h) Images of the tilted sample after being rotated and scaled back to their circular form, respectively. (i) The process for converting a tilted image into its corresponding perpendicular view: the algorithm detects the hydrophobic barrier in the captured image, aligns the principal axes of the extracted ellipse to the horizontal and vertical directions via rotation, and finally scales the ellipse back into its original circular shape, enabling downstream analysis for sample screening and concentration characterization. For enhanced clarity, microscopic images were utilized for the zoomed-in views.

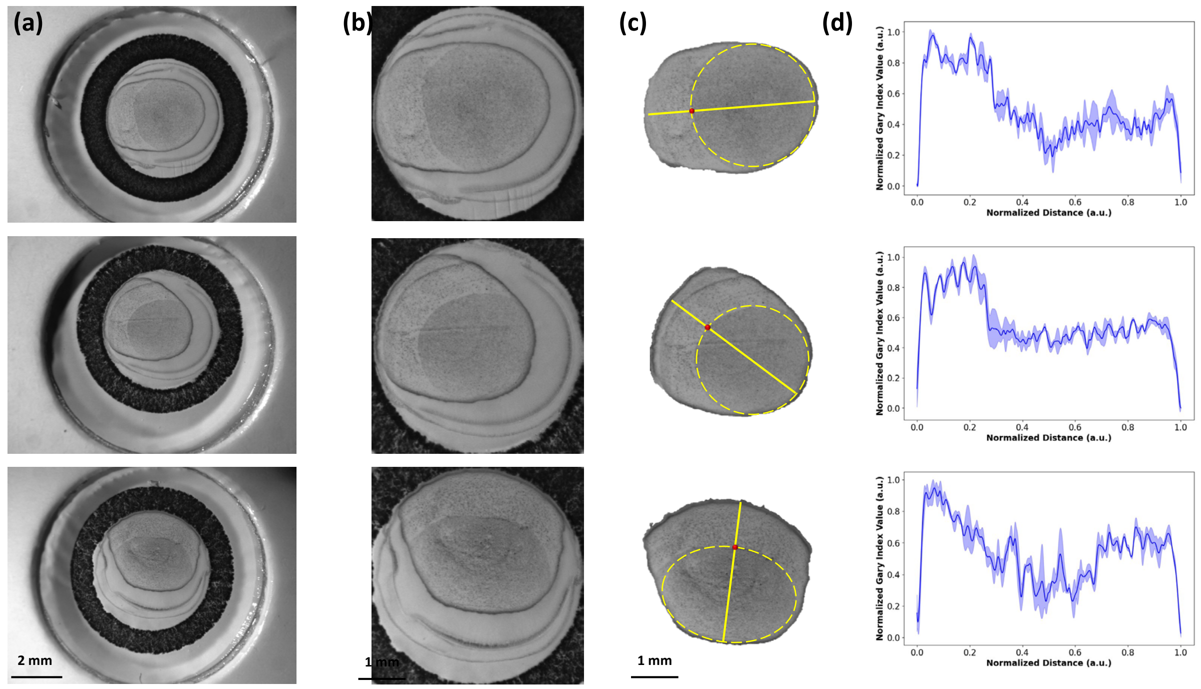

Figure.S27: Coffee-ring detection examples. (a) Captured optical photos for three PSA-spiked samples at a 1 ng/ml concentration. (b) Zoomed-in views of the detection zone. (c) C-GAN–based specific zone segmentation for the crossline position and gray index value extraction. (d) Extracted crossline profiles showing similar features across three different patterns with the same 1 ng/ml concentration with good consistency. The calibration process help mitigating slight variations in crossline profiles by checking main features of multiple crosslines using the trained FC network. Note, results are expressed as mean values ± standard deviation, based on n = 3 sample lines.

References

[1] R. Gupta *et al.*, "Ultrasensitive lateral-flow assays via plasmonically active antibody-conjugated fluorescent nanoparticles," *Nat. Biomed. Eng.*, vol. 7, no. 12, pp. 1556–1570, 2023, doi: 10.1038/s41551-022-01001-1.

[2] H. Cho *et al.*, "Electrochemical Impedance-Based Biosensors for the Label-Free Detection of the Nucleocapsid Protein from SARS-CoV-2," *ACS Sensors*, vol. 7, no. 6, pp. 1676–1684, 2022, doi: 10.1021/acssensors.2c00317.

[3] D. R. P. Nicollete *et al.*, "Enhancing a SARS-CoV-2 nucleocapsid antigen test sensitivity with cost efficient strategy through a cotton intermembrane insertion," *Sci. Rep.*, vol. 13, no. 1, pp. 1–13, 2023, doi: 10.1038/s41598-023-31641-5.

[4] L. Shen *et al.*, "A rapid lateral flow immunoassay strip for detection of SARS-CoV-2 antigen using latex microspheres," *J. Clin. Lab. Anal.*, vol. 35, no. 12, pp. 1–7, 2021, doi: 10.1002/jcla.24091.

[5] S. M. Schubert *et al.*, "Ultra-sensitive protein detection via Single Molecule Arrays towards early stage cancer monitoring," *Sci. Rep.*, vol. 5, pp. 1–8, 2015, doi: 10.1038/srep11034.

[6] A. Raziq, A. Kidakova, R. Boroznjak, J. Reut, A. Öpik, and V. Syritski, "Development of a portable MIP-based electrochemical sensor for detection of SARS-CoV-2 antigen," *Biosens. Bioelectron.*, vol. 178, p. 113029, 2021, doi: https://doi.org/10.1016/j.bios.2021.113029.

[7] F. Haghayegh, R. Salahandish, M. Hassani, and A. Sanati-Nezhad, "Highly Stable Buffer-Based Zinc Oxide/Reduced Graphene Oxide Nanosurface Chemistry for Rapid Immunosensing of SARS-CoV-2 Antigens," *ACS Appl. Mater. Interfaces*, vol. 14, no. 8, pp. 10844–10855, Mar. 2022, doi: 10.1021/acsami.1c24475.

[8] D. Soto and J. Orozco, "Peptide-based simple detection of SARS-CoV-2 with electrochemical readout," *Anal. Chim. Acta*, vol. 1205, p. 339739, 2022, doi: https://doi.org/10.1016/j.aca.2022.339739.

[9] A. Yakoh, U. Pimpitak, S. Rengpipat, N. Hirankarn, O. Chailapakul, and S. Chaiyo, "Paper-based electrochemical biosensor for diagnosing COVID-19: Detection of SARS-CoV-2 antibodies and antigen," *Biosens. Bioelectron.*, vol. 176, p. 112912, 2021, doi: https://doi.org/10.1016/j.bios.2020.112912.

[10] R. Funari, K.-Y. Chu, and A. Q. Shen, "Detection of antibodies against SARS-CoV-2 spike protein by gold nanospikes in an opto-microfluidic chip," *Biosens. Bioelectron.*, vol. 169, p. 112578, 2020, doi: https://doi.org/10.1016/j.bios.2020.112578.

[11] N. K. Singh *et al.*, "Hitting the diagnostic sweet spot: Point-of-care SARS-CoV-2 salivary antigen testing with an off-the-shelf glucometer," *Biosens. Bioelectron.*, vol. 180, p. 113111, 2021, doi: https://doi.org/10.1016/j.bios.2021.113111.

[12] G. Seo *et al.*, "Rapid Detection of COVID-19 Causative Virus (SARS-CoV-2) in Human Nasopharyngeal Swab Specimens Using Field-Effect Transistor-Based Biosensor," *ACS Nano*, vol. 14, no. 4, pp. 5135–5142, 2020, doi: 10.1021/acsnano.0c02823.

[13] M. S. Draz *et al.*, "Virus detection using nanoparticles and deep neural network–enabled smartphone system," *Sci. Adv.*, vol. 6, no. 51, p. eabd5354, Dec. 2023, doi: 10.1126/sciadv.abd5354.

[14] S. Boonkaew, K. Szot-Karpińska, J. Niedziółka-Jönsson, A. de Marco, and M. Jönsson-Niedziółka, "NFC Smartphone-Based Electrochemical Microfluidic Device Integrated with Nanobody Recognition for C-Reactive Protein," *ACS Sensors*, vol. 9, no. 6, pp. 3066–3074, Jun. 2024, doi: 10.1021/acssensors.4c00249.

[15] A. M. Jankelow *et al.*, “Smartphone clip-on instrument and microfluidic processor for rapid sample-to-answer detection of Zika virus in whole blood using spatial RT-LAMP,” *Analyst*, vol. 147, no. 17, pp. 3838–3853, 2022, doi: 10.1039/D2AN00438K.

[16] J. Su *et al.*, “Smartphone-Based Electrochemical Biosensors for Directly Detecting Serum-Derived Exosomes and Monitoring Their Secretion,” *Anal. Chem.*, vol. 94, no. 7, pp. 3235–3244, Feb. 2022, doi: 10.1021/acs.analchem.1c04910.

[17] Z. Tang *et al.*, “SLIDE: Saliva-Based SARS-CoV-2 Self-Testing with RT-LAMP in a Mobile Device,” *ACS Sensors*, vol. 7, no. 8, pp. 2370–2378, Aug. 2022, doi: 10.1021/acssensors.2c01023.

[18] Z. Yuan *et al.*, “A cost-effective smartphone-based device for rapid C-reaction protein (CRP) detection using magnetoelastic immunosensor,” *Lab Chip*, vol. 23, no. 8, pp. 2048–2056, 2023, doi: 10.1039/D2LC01065H.

[19] S. Dutta, K. Saikia, and P. Nath, “Smartphone based LSPR sensing platform for bio-conjugation detection and quantification,” *RSC Adv.*, vol. 6, no. 26, pp. 21871–21880, 2016, doi: 10.1039/C6RA01113F.

[20] K. Simonyan and A. Zisserman, “Very Deep Convolutional Networks for Large-Scale Image Recognition,” *arXiv*, 2014.

[21] O. Ronneberger, P. Fischer, and T. Brox, “U-Net: Convolutional Networks for Biomedical Image Segmentation,” in *Medical Image Computing and Computer-Assisted Intervention – MICCAI*, 2015, pp. 234–241, doi: 10.48550/arXiv.1505.04597.

[22] P. Isola, J.-Y. Zhu, T. Zhou, and A. A. Efros, “Image-to-image translation with conditional adversarial networks,” in *Proceedings of the IEEE conference on computer vision and pattern recognition*, 2017, pp. 1125–1134, doi: 10.1109/CVPR.2017.632.

[23] Y. Luo *et al.*, “Component identification for the SERS spectra of microplastics mixture with convolutional neural network,” *Sci. Total Environ.*, vol. 895, p. 165138, 2023, doi: https://doi.org/10.1016/j.scitotenv.2023.165138.

[24] A. Borji, “Pros and cons of GAN evaluation measures,” *Comput. Vis. Image Underst.*, vol. 179, no. February 2018, pp. 41–65, 2019, doi: 10.1016/j.cviu.2018.10.009.

[25] N. Elaraby, S. Barakat, and A. Rezk, “A conditional GAN-based approach for enhancing transfer learning performance in few-shot HCR tasks,” *Sci. Rep.*, vol. 12, no. 1, p. 16271, 2022, doi: 10.1038/s41598-022-20654-1.

[26] V. Paranthaman *et al.*, “Experimental and theoretical insights into enhanced light harvesting in dye-sensitized solar cells via Au@TiO2 core-shell and BaTiO3 nanoparticles,” *J. Taiwan Inst. Chem. Eng.*, vol. 165, p. 105778, 2024, doi: https://doi.org/10.1016/j.jtice.2024.105778.

[27] M. Skiba, L. Khrokalo, A. Linyuchev, and V. Vorobyova, “State of the art on plasma-liquid route synthesis monometallic Au, bimetallic Au/Ag core–shell and alloy nanoparticles: Toxicological, canalytical, antimicrobial activity for environmental control,” *J. Mol. Struct.*, vol. 1318, p. 139293, 2024, doi: https://doi.org/10.1016/j.molstruc.2024.139293.

[28] X. Shen, C.-M. Ho, and T.-S. Wong, “Minimal Size of Coffee Ring Structure,” *J. Phys. Chem. B*, vol. 114, no. 16, pp. 5269–5274, Apr. 2010, doi: 10.1021/jp912190v.
