# Supplementary material for: Plasmonic coffee-ring biosensing for AI-assisted point-of-care diagnostics
Source: Nat Commun. 2025 May 17;16:4597. doi: 10.1038/s41467-025-59868-y (PMC12085679; doi:10.1038/s41467-025-59868-y)
Supplement: Supplementary file 2 — Description of Additional Supplementary Files [file 41467_2025_59868_MOESM2_ESM.pdf]

Description of Additional Supplementary Files

Supplementary Movie 1: The evaporation steps of a plasmonic droplet. The process includes four stages: 1- Spreading, 2- Fixed contact radius evaporation, 3- Fixed contact angle evaporation, and 4- Backward evaporation through membrane. The asymmetric plasmonic pattern provides insights into the specific protein content of the sample.
