# Supplementary material for: Plasmonic coffee-ring biosensing for AI-assisted point-of-care diagnostics
Source: Nat Commun. 2025 May 17;16:4597. doi: 10.1038/s41467-025-59868-y (PMC12085679; doi:10.1038/s41467-025-59868-y)
Supplement: Supplementary file 4 — Reporting Summary [file 41467_2025_59868_MOESM4_ESM.pdf]

nature portfolio

Corresponding author(s): Kamyar Behrouzi, Liwei Lin

Last updated by author(s): 4/12/2025

Reporting Summary

Nature Portfolio wishes to improve the reproducibility of the work that we publish. This form provides structure for consistency and transparency in reporting. For further information on Nature Portfolio policies, see our Editorial Policies and the Editorial Policy Checklist.

Statistics

For all statistical analyses, confirm that the following items are present in the figure legend, table legend, main text, or Methods section.

n/a	Confirmed	
☐	☒	The exact sample size (*n*) for each experimental group/condition, given as a discrete number and unit of measurement
☐	☒	A statement on whether measurements were taken from distinct samples or whether the same sample was measured repeatedly
☒	☐	The statistical test(s) used AND whether they are one- or two-sided<br>*Only common tests should be described solely by name; describe more complex techniques in the Methods section.*
☒	☐	A description of all covariates tested
☐	☒	A description of any assumptions or corrections, such as tests of normality and adjustment for multiple comparisons
☐	☒	A full description of the statistical parameters including central tendency (e.g. means) or other basic estimates (e.g. regression coefficient) AND variation (e.g. standard deviation) or associated estimates of uncertainty (e.g. confidence intervals)
☒	☐	For null hypothesis testing, the test statistic (e.g. *F*, *t*, *r*) with confidence intervals, effect sizes, degrees of freedom and *P* value noted<br>*Give P values as exact values whenever suitable.*
☒	☐	For Bayesian analysis, information on the choice of priors and Markov chain Monte Carlo settings
☒	☐	For hierarchical and complex designs, identification of the appropriate level for tests and full reporting of outcomes
☒	☐	Estimates of effect sizes (e.g. Cohen's *d*, Pearson's *r*), indicating how they were calculated

Our web collection on statistics for biologists contains articles on many of the points above.

Software and code

Policy information about availability of computer code

Data collection	No software was used for data collection.
Data analysis	Google CoLab for all codings, ImageJ for Lateral flow intensity measurements, Python 3.10 for data analysis and plotting, Tensorflow 2.16.1 for machine learning

For manuscripts utilizing custom algorithms or software that are central to the research but not yet described in published literature, software must be made available to editors and reviewers. We strongly encourage code deposition in a community repository (e.g. GitHub). See the Nature Portfolio guidelines for submitting code & software for further information.

Data

Policy information about availability of data

All manuscripts must include a data availability statement. This statement should provide the following information, where applicable:

- Accession codes, unique identifiers, or web links for publicly available datasets
- A description of any restrictions on data availability
- For clinical datasets or third party data, please ensure that the statement adheres to our policy

The data that support the plots and other findings of this study are available from the corresponding authors upon reasonable request.

Research involving human participants, their data, or biological material

Policy information about studies with human participants or human data. See also policy information about sex, gender (identity/presentation), and sexual orientation and race, ethnicity and racism.

Reporting on sex and gender	The demographic information of the donors is not tracked or stated for the pooled material, unless requested at the time of the order. The material is collected from consented donors. Stated by Innovative Research company.
Reporting on race, ethnicity, or other socially relevant groupings	The demographic information of the donors is not tracked or stated for the pooled material, unless requested at the time of the order. The material is collected from consented donors. Stated by Innovative Research company.
Population characteristics	The demographic information of the donors is not tracked or stated for the pooled material, unless requested at the time of the order. The material is collected from consented donors. Stated by Innovative Research company.
Recruitment	Participants are compensated under IRB. Stated by Innovative Research company.
Ethics oversight	Ethical oversight was conducted under the IRB. Stated by Innovative Research company.

Note that full information on the approval of the study protocol must also be provided in the manuscript.

Field-specific reporting

Please select the one below that is the best fit for your research. If you are not sure, read the appropriate sections before making your selection.

- [x] Life sciences
- [ ] Behavioural & social sciences
- [ ] Ecological, evolutionary & environmental sciences

For a reference copy of the document with all sections, see nature.com/documents/nr-reporting-summary-flat.pdf

Life sciences study design

All studies must disclose on these points even when the disclosure is negative.

Sample size	Protein sensing experiments were done using our own developed coffee-ring biosensor and all the information about the sample size and number of measurements were given in the main manuscript.
Data exclusions	No data were excluded.
Replication	The protein binding experiments were reproducible and measured across a large number of individual coffee-ring biosensors, repeated at least three times for each case. Data are included in the paper.
Randomization	No randomization was used because the studies utilized purified protein samples to demonstrate sensor performance rather than comparing clinical samples. Saliva specimens were pooled to provide a background matrix for protein target detection. Additionally, we used uniform data collection and consistent statistical analysis throughout the research. It should be noted, Note, no distinct treatments, interventions, or procedures, such as those typical in clinical trials, were performed on different groups of samples.
Blinding	Blinding was not applicable to this study as protein targets were from purified materials. Note, no distinct treatments, interventions, or procedures, such as those typical in clinical trials, were performed on different groups of samples.

Reporting for specific materials, systems and methods

We require information from authors about some types of materials, experimental systems and methods used in many studies. Here, indicate whether each material, system or method listed is relevant to your study. If you are not sure if a list item applies to your research, read the appropriate section before selecting a response.

Materials & experimental systems

n/a	Involved in the study	
[ ]	[x]	Antibodies
[x]	[ ]	Eukaryotic cell lines
[x]	[ ]	Palaeontology and archaeology
[x]	[ ]	Animals and other organisms
[x]	[ ]	Clinical data
[x]	[ ]	Dual use research of concern
[x]	[ ]	Plants

Methods

n/a	Involved in the study	
[x]	[ ]	ChIP-seq
[x]	[ ]	Flow cytometry
[x]	[ ]	MRI-based neuroimaging

Antibodies

Antibodies used	From Sino Biological<br>1- Anti-Procalcitonin/CALCA Antibody, Rabbit Polyclonal, Cat: 13933-T16<br>2- Anti-CEACAM-3/CD66d Antibody, Rabbit Polyclonal, Cat: 11933-T24<br>3- SARS-CoV-2 (2019-nCoV) Nucleocapsid Antibody, Rabbit PAb, Antigen Affinity Purified, Cat: 40588-T62<br>4- Anti-KLK3/PSA Antibody, Rabbit Polyclonal, Cat: 10771-RP02
Validation	See the provider website:<br>1- https://www.sinobiological.com/antibodies/human-procalcitonin-calca-13933-t16<br>2- https://www.sinobiological.com/antibodies/human-ceacam3-11933-t24<br>3- https://www.sinobiological.com/antibodies/nucleocapsid-np-40588-t62<br>4- https://www.sinobiological.com/antibodies/human-klk3-10771-rp02

Plants

Seed stocks	N/A
Novel plant genotypes	N/A
Authentication	N/A
